# Peripheral vaccination-induced brain-resident memory CD8^+^ T cells durably protect mice against intracranial malignancy

**DOI:** 10.1172/JCI197812

**Published:** 2026-04-15

**Authors:** Madison R. Mix, Cassie M. Sievers, Mariah Hassert, Shravan Kumar Kannan, Lecia L. Pewe, Sunny C. Huang, Rui He, Cori E. Fain, Mohammad Heidarian, Lisa S. Hancox, Sahaana A. Arumugam, Terry G. Beltz, Fang Jin, Aaron J. Johnson, Calvin S. Carter, Noah S. Butler, Aliasger K. Salem, Vladimir P. Badovinac, John T. Harty

**Affiliations:** 1 Department of Pathology, Carver College of Medicine,; 2 Medical Scientist Training Program, Carver College of Medicine,; 3 Interdisciplinary Graduate Program in Immunology, Carver College of Medicine,; 4 Holden Comprehensive Cancer Center, Carver College of Medicine,; 5 Free Radical and Radiation Biology Training Program, Department of Radiation Oncology, Carver College of Medicine,; 6 Department of Pharmaceutical Sciences and Experimental Therapeutics, College of Pharmacy,; 7 Experimental Pathology Graduate Program, Carver College of Medicine, and; 8 Department of Neuroscience and Pharmacology, Carver College of Medicine, University of Iowa, Iowa City, Iowa, USA.; 9 Department of Immunology, Mayo Clinic, Rochester, Minnesota, USA.; 10 Department of Microbiology and Immunology, Carver College of Medicine, University of Iowa, Iowa City, Iowa, USA.

**Keywords:** Immunology, Neuroscience, Oncology, Memory, T cells

## Abstract

Primary and metastatic brain tumors exhibit resistance to immunotherapies that demonstrate efficacy in peripheral cancer settings. While many immunotherapies aim to enhance CD8^+^ T cell infiltration and functionality in established tumors, identification of neoantigens support emerging immunopreventative tactics against brain cancer. Functionally potent tissue-resident memory CD8^+^ T cells (T_RM_) can be generated in the brain following peripheral infection or vaccination. However, the ability of brain T_RM_ to prevent intracranial malignancy remains unknown. Here, mice were seeded with tumor-specific or bystander brain T_RM_ via peripheral infection prior to depletion of circulating memory T cells (T_CIRCM_) and subsequent brain tumor challenge. Tumor-specific brain T_RM_ durably protected mice against intracranial malignancy even in the absence T_CIRCM_. These brain T_RM_ persisted in tumor-surviving mice and protected against a second antigen-matched challenge. Importantly, a translationally-relevant mRNA-lipid nanoparticle (LNP) vaccine phenocopied peripheral infection-induced outcomes, generating functional brain T_RM_ that controlled tumor growth. Altogether, this work points to the utility of brain T_RM_ in cancer immunoprevention, supporting the development of antitumor mRNA-LNP vaccines to bolster brain immunity.

## Abstract 2

Mix et al. demonstrate that the generation of anti-tumor brain-resident memory CD8^+^ T cells via peripheral vaccination can significantly attenuate adverse outcomes associated with brain malignancy.

## Graphical abstract

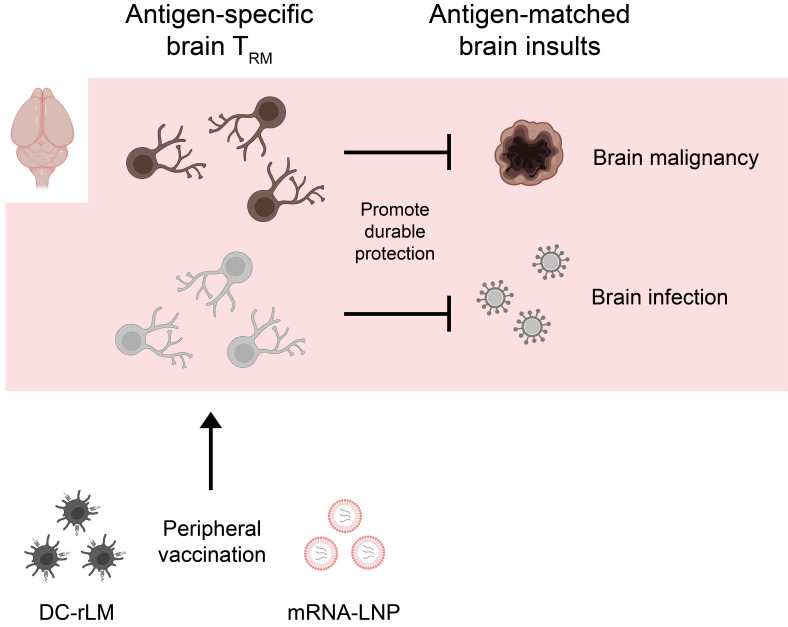

## Introduction

Primary and metastatic brain tumors pose substantial challenges to positive therapeutic outcomes. Collectively, the brain’s (a) spatial encasement within the skull, (b) vasculature-based barriers, (c) limited regenerative potential, (d) glymphatic/meningeal drainage systems, and (e) capacity to induce local inflammation and peripheral immunosuppression establish obstacles against cancer therapies that are efficacious in other organs (1–8). Unfortunately, these hurdles have not disappeared with the emergence of cancer immunotherapies. While designed to enhance CD8^+^ T cell presence and functionality in established tumors, checkpoint blockade, adoptive T cell/dendritic cell vaccination, and chimeric antigen receptor (CAR) T cell–based therapies have not substantially enhanced 5-year survival rates among most brain cancer patients (9–14).

Due to historical conceptions of immune privilege, the study of CD8^+^ T cell biology in the brain remains in its infancy (15). In humans, antigen-experienced, memory CD8^+^ T cells populate the healthy and tumor-affected brain, correlating positively with cancer prognosis (16–24). Unlike circulating memory T cells (T_CIRCM_) that continuously surveil the body, tissue-resident memory (T_RM_) T cells embed permanently in tissues like the brain (15, 25). Brain T_RM_ express markers such as CD69, CD49a, and CXCR6 to promote residency and are poised to quickly wield protective effector functions upon antigen reencounter (17, 26–31). Recent studies have revealed a subset of T_RM_ in human brain tumors that are specific for viral pathogens, such as cytomegalovirus, Epstein-Barr virus, and influenza virus (17). While viral peptide-based reactivation of pathogen-specific memory T cells reduces malignant outcomes in peripheral tumors, brain-localized tumors exhibit greater resistance to this approach (17, 32). In parallel, tumor-specific memory T cells exist within brain cancer microenvironments. Recent literature, however, underscores that T_RM_ signatures reported among CD8^+^ T cells isolated from tumors may fail to identify true functional, resident T cells (33). Here, antigen-rich tumors, unlike healthy tissue, impart a distinct program of tissue residency that hinders the functionality of newly generated T_RM_-appearing cells, pointing to potential difficulties in rejuvenating these cell populations (33, 34).

While cancer treatments have historically been therapeutic, emerging technologies are increasing the ability to identify tumor neoantigens and leverage immunopreventative approaches (35–39). Tumor-specific T_RM_ generated in healthy cutaneous and mucosal tissues prior to tumor challenge offer prolonged protection against malignancy in mice (40–43). In these investigations and others, the generation of T_RM_ in barrier tissues (i.e., skin, lung, intestine) and vascularly accessible tissues (i.e., liver) is magnified when the organ of interest is directly infected, immunized, or inflamed (40–46). While brain T_RM_ can be engendered by neurotropic infection or intracranial vaccination, immunogenic agent administration directly to brain tissue may not represent a clinically or surgically palatable strategy (15). In spite of this, emerging work has demonstrated that peripheral infections and immunizations are capable of generating brain T_RM_, offering hope that translational platforms can assemble these brain-resident populations (15, 27, 30). Peripherally-induced brain T_RM_ have been shown to protect against otherwise lethal intracranial infections, demonstrating their functional potency when seeded in healthy brain tissue (27). However, whether peripherally induced, tumor-specific T_RM_ can defend against imminent brain tumorigenesis remains unknown.

The clinical viability of cancer immunoprevention is growing (35, 47). Like pathogens, tumors harbor targetable antigens that can be identified and implemented into vaccine regimens (24, 37, 39). Emerging immunization platforms are enabling the rapid and cost-effective generation of personalized anticancer vaccines. Patient-tailored, neoantigen-based mRNA vaccines can be designed and administered within a timeframe of 9 weeks, hastening a new era of immunotherapy for patients diagnosed with circulating or solid tumors (37). In patients with pancreatic cancer, mRNA vaccines have been shown to generate long-lived CD8^+^ T cells with memory-like phenotypes (48). Emerging evidence also suggests mRNA vaccination may help control or prevent metastases in distant solid organs (37). Therefore, vaccination efforts could be particularly relevant for (a) patients with peripheral cancers that have not metastasized, (b) patients with primary cancers with limited spread within a given tissue, (c) patients in cancer remission, and (d) patients harboring genetic predispositions to cancer (35, 47).

The underlying immunology of mRNA vaccines, particularly with respect to T_RM_ biology in the brain, is difficult to determine in human patients, necessitating studies in tractable animal models. mRNA vaccination has already been shown to establish and regenerate T_RM_ populations in tissues like the lung and liver in mice (44–46, 49). However, the brain and its related tumors represent uncharted territory with respect to mRNA vaccine technology (50). The seeding of tumor-specific T_RM_ in healthy brain tissue represents an unexplored conceptual approach to thwart future intracranial tumor establishment or spread. Therein, we generated mice with tumor-specific brain T_RM_ via peripheral infection or mRNA vaccination and tested the potency of these cells against brain tumor challenges.

## Results

### Tumor-specific brain T_RM_ durably protect against intracranial malignancy.

**Figure 1:**
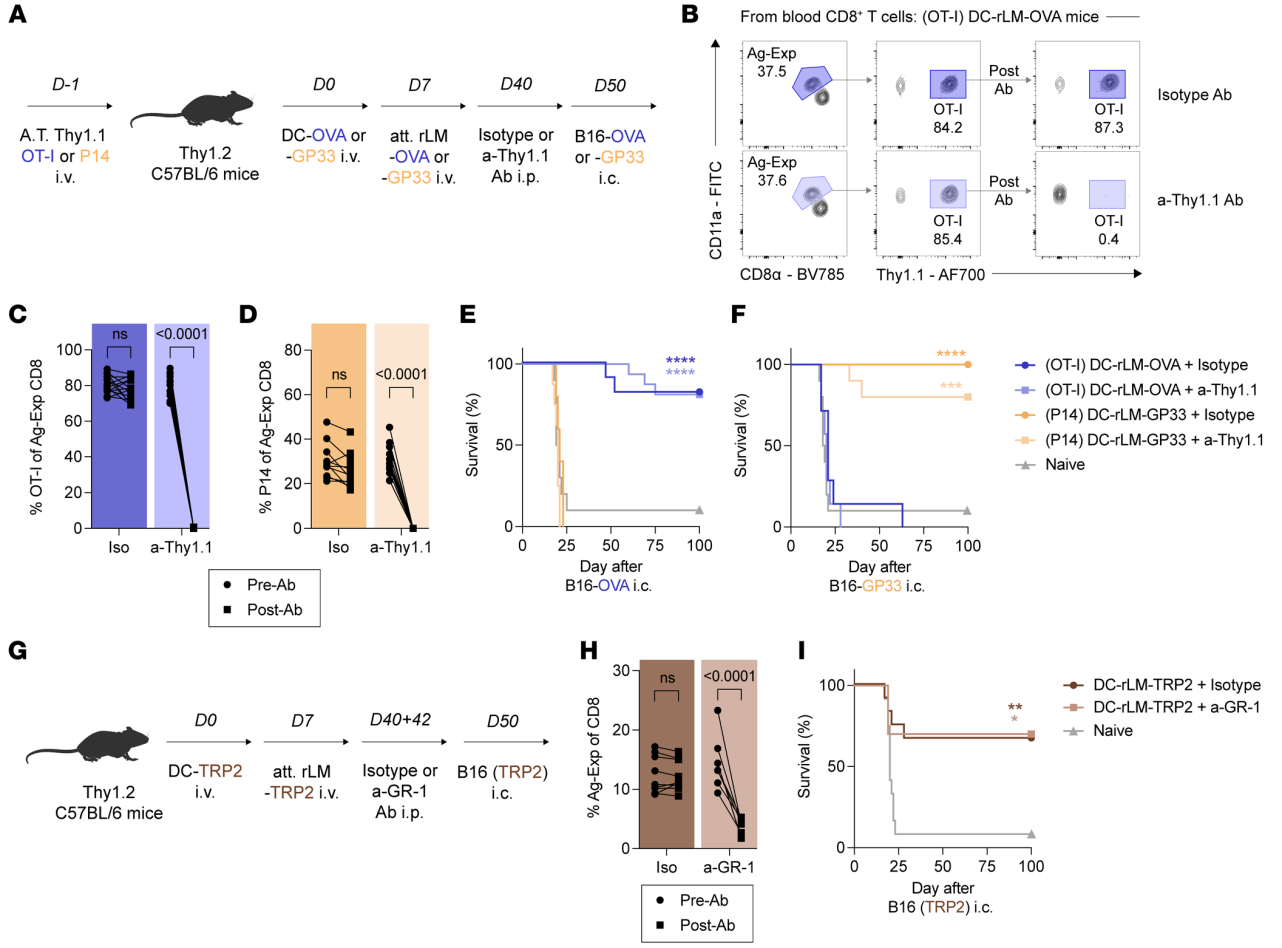
Peripherally induced, tumor-specific brain T_RM_ provide durable protection against intracranial melanoma. (**A**) Experimental design. Thy1.2 C57BL/6N mice were adoptively transferred with 10^4^ Thy1.1 OT-I or 2 × 10^4^ Thy1.1 P14 intravenously (i.v.) 1 day before i.v. injection with OVA_257–264_ or GP_33–41_ peptide-pulsed, LPS-matured dendritic cells (DC-OVA and DC-GP33). Mice were boosted with attenuated recombinant *Listeria monocytogenes* expressing OVA_257–264_ or GP_33–41_ (att. rLM-OVA and rLM-GP33). Mice were treated with 2 μg isotype control or a-Thy1.1 antibody (Ab) intraperitoneally (i.p.) to deplete OT-I or P14 T_CIRCM_ before intracranial (i.c.) challenge with B16-F10 melanoma cells expressing OVA_257–264_ or GP_33–41_ (B16-OVA and B16-GP33). (**B**) OT-I cells among CD11a^hi^, antigen-experienced (Ag-Exp) T_CIRCM_ in the blood pre/post-Ab depletion. (**C**) Frequency of OT-I and (**D**) P14 of Ag-Exp CD8^+^ T cells in the blood pre/post-Ab depletion. (**E**) Kaplan-Meier survival curves after B16-OVA or (**F**) B16-GP33 i.c. challenge. (**G**) Experimental design. C57BL/6N mice were injected with TRP2_180–188_ peptide-pulsed, LPS-matured DCs (DC-TRP2) i.v. and boosted with att. rLM-TRP2. Mice were treated with 200 μg isotype control or a-GR-1 Ab i.p. to deplete endogenous CD8^+^ T_CIRCM_ before i.c. challenge with B16-F10 cells. (**H**) Frequency of Ag-Exp cells among CD8^+^ T cells in blood pre/post-Ab depletion. (**I**) Kaplan-Meier survival curves of mice after B16 i.c. challenge. Experiments (**E**, **F**, and **I**) show concatenated data from 3 independent experiments with *n* = 5–16 mice per group. Experiments (**B**–**D** and **H**) show concatenated data from 2–3 independent experiments with *n* = 7–12 mice per group. Statistical significance was determined by paired *t* test or log-rank test for survival curves with comparison with naive mice. Graphs show the mean ± SEM with each symbol representing 1 mouse. Individual *P* values are noted on respective graphs or are otherwise summarized as: *P < 0.05, **P < 0.01, ***P < 0.001, ****P < 0.0001. Graphical illustrations were created using BioRender (https://biorender.com).

Our group has previously shown that peripheral infections and immunizations in mice can generate pathogen-specific brain T_RM_ that protect against subsequent intracranial infection (27, 30). Here, we leveraged these published, peripheral immunization approaches to produce brain T_RM_ populations with specificity to tumor cells. Accordingly, we adoptively transferred Thy1.2 C57BL/6N (B6) mice with physiologic numbers of allelically disparate Thy1.1 T cell receptor transgenic (TCR-tg) CD8^+^ T cells (Figure 1A). These TCR-tg cells were either OT-I cells specific for the OVA_257–264_ peptide derived from chicken ovalbumin or P14 cells specific for the GP_33–41_ peptide derived from lymphocytic choriomeningitis virus (LCMV). Recipient mice were then intravenously (i.v.) injected with peptide-pulsed, LPS-matured dendritic cells (DC) and boosted 7 days later with recombinant, attenuated *Listeria monocytogenes* (rLM) expressing OVA_257–264_ or GP_33–41_ (DC-rLM-OVA and DC-rLM-GP33) i.v. (27, 51). At day 40 post-DC immunization, mice were treated intraperitoneally (i.p.) with a low-dose isotype control or anti-Thy1.1 (a-Thy1.1) depleting antibodies. We have previously published that this a-Thy1.1 antibody regimen effectively depletes OT-I/P14 T_CIRCM_ while preserving T_RM_ populations in the brain (27). Here, we validated that this antibody regimen depletes Thy1.1^+^ T cells in brain-adjacent tissues such as the cranial bone and meninges in addition to distant nonlymphoid organs such as the liver, kidney, and lung (Supplemental Figure 1, A–C; supplemental material available online with this article; https://doi.org/10.1172/JCI197812DS1). In these experiments, mice were injected i.v. with a fluorochrome-conjugated anti-CD45 antibody to distinguish cells in the vasculature (IV^+^) versus cells localized in tissues (IV^–^) at the time of tissue harvest (52). Successful depletion of OT-I / P14 T_CIRCM_ among antigen-experienced (Ag-Exp), CD11a^hi^ memory CD8^+^ T cells in the blood was also confirmed in mice prior to each tumor challenge (Figure 1, B–D).

Metastatic brain tumors are 10 times more common than primary brain tumors and develop in 10%–30% of cancer patients (53, 54). Of these metastatic tumors, lung, breast, and melanoma represent the most common primary origins (53, 54). Melanoma tumors commonly metastasize to the brain and can be modeled orthotopically in mice (1, 2, 55, 56). While lacking the spatial and temporal realism of spontaneously metastasizing models or intravenous/intracarotid tumor injections, orthotopic injections provide conceptual and technical feasibility for interrogating T_RM_-based protection independent of peripheral disease. Accordingly, we challenged naive and immune mice intracranially (i.c.) with B16 melanoma cells expressing OVA_257–264_ (B16-OVA) or GP_33–41_ (B16-GP33). Here, mice with OT-I brain T_RM_, with or without T_CIRCM_, exhibited a substantial survival advantage over naive mice after challenge with B16-OVA, but not B16-GP33 (Figure 1, E and F). Reciprocal results were achieved in mice containing P14 brain T_RM_ (Figure 1, E and F). Thus, we demonstrate that tumor-specific, but not bystander, brain T_RM_ provide durable protection against intracranial B16 melanoma even in the absence of T_CIRCM_. These data indicate that the formation of tumor-specific brain T_RM_ in healthy brain tissue can provide superior protection than de novo CD8^+^ T cell responses, preventing imminent malignancy in a large proportion of challenged hosts.

While the prior studies provided insight leveraging TCR-tg CD8^+^ T cells and model epitopes, we wished to implement a more realistic model system where protective outcomes could be assessed based on immunization against antigens, such as TRP2_180–188_, expressed by melanocytic cells and tumors. Therefore, we extended our studies to determine the ability of endogenous, tumor-specific brain T_RM_ to protect against intracranial malignancy. Here, mice were primed and boosted with DC-rLM expressing the B16-derived epitope TRP2_180–188_ (Figure 1G) (57). To broadly deplete T_CIRCM_ encompassing TRP2_180–188_-specific populations, we leveraged an established antibody depletion strategy that capitalizes on the expression of Ly6C among CD8^+^ T_CIRCM_, representing a marker not expressed by T_RM_ (58). Accordingly, we treated mice with 2 doses of a-GR-1 antibody to deplete Ly6C/G^+^ immune cells prior to tumor challenge. We observed a substantial reduction in the proportion of CD11a^hi^ T_CIRCM_ among total CD8^+^ T cells in the blood following a-GR-1 antibody treatment (Figure 1H and Supplemental Figure 2A). Importantly, we did not observe a proportional reduction of CD11a^hi^ T_RM_ in the brain, including those specific for TRP2_180–188_, as confirmed by tetramer staining (Supplemental Figure 2, B–D). In these immunized mice, we demonstrate approximately 65% long-term survival against an intracranial B16 melanoma challenge, even in the absence of T_CIRCM_ (Figure 1I). Together, these data demonstrate that the generation of brain T_RM_ targeting an endogenous melanoma epitope can durably improve survival outcomes against intracranial malignancy.

### Brain T_RM_ persist long term in tumor-surviving mice.

**Figure 2:**
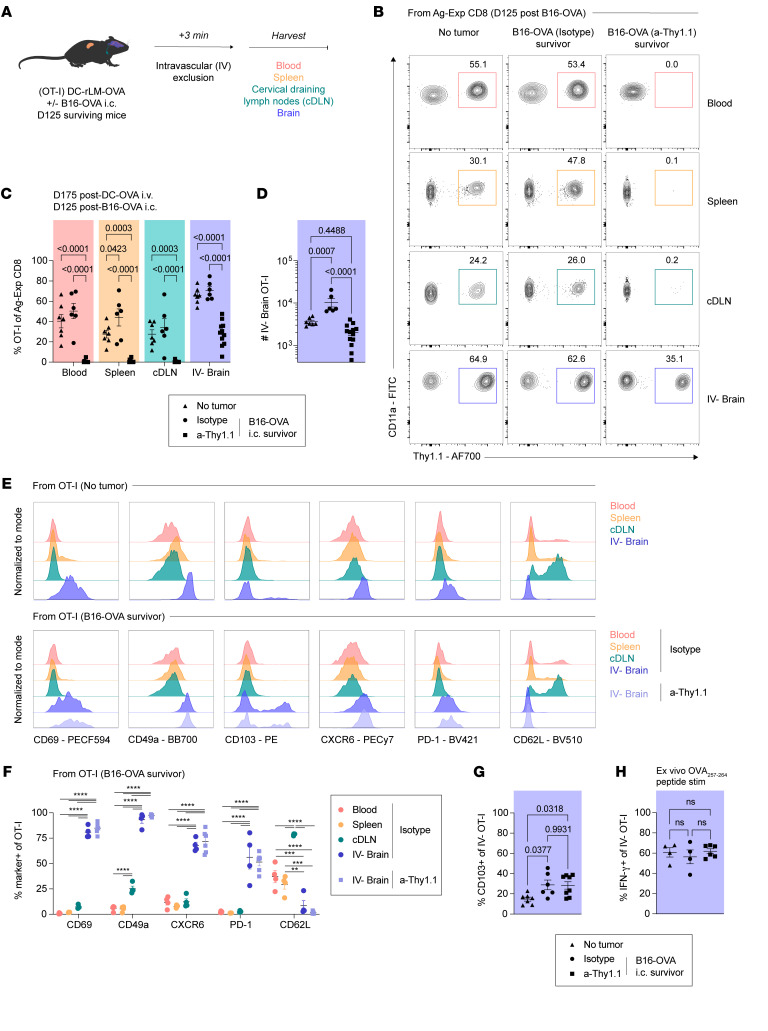
T_RM_ persist in the brain following intracranial tumor challenge. (**A**) Experimental design. At 125 days post-B16-OVA challenge, tumor-surviving mice with prior antibody depletion regimens from Figure 1 or age-matched, immunized, tumor-naive mice were intravenously (IV) injected with a fluorophore-conjugated anti-CD45 antibody approximately 3 minutes before tissue harvest. Blood, spleen, cervical draining lymph nodes (cDLN), and brain tissues were isolated. (**B**) Representative flow plots and (**C**) frequencies of OT-I in the blood, spleen, cDLN, and IV^–^ brain of tumor naive, tumor surviving isotype-treated mice, and tumor surviving a-Thy1.1-treated mice. (**D**) Number of OT-I in the IV^–^ brain. (**E**) Representative histograms of T_RM_-associated markers among OT-I in tumor naive and antibody-treated tumor-surviving mice across tissue compartments. (**F**) Frequency of T_RM_-associated marker expression in isotype or a-Thy1.1 antibody-treated tumor-surviving mice. (**G**) Frequency of CD103^+^ IV^–^ brain OT-I. (**H**) Frequency of IFN-γ^+^ OT-I in the IV^–^ brain following 5–6-hour ex vivo 1 μM OVA_257–264_ peptide stimulation. Experiments (**A**–**D**) show concatenated data from 3 independent experiments with *n* = 6–11 mice per group total. Experiments (**F** and **H**) show representative data from 1 of 2 independent experiments with *n* = 4–6 mice per group. Experiments (**G**) show concatenated data from 2 independent experiments with *n* = 6–8 mice per group. Statistical significance was determined by 1-way ANOVA with Tukey’s multiple comparison’s test. Graphs show the mean ± SEM with each symbol representing 1 mouse. Individual *P* values are noted on respective graphs or are otherwise summarized as: *P < 0.05, **P < 0.01, ***P < 0.001, ****P < 0.0001. Graphical illustrations were created using BioRender (https://biorender.com).

Our previous experiments presented the unique opportunity to study outcomes derived from brain tumor–surviving mice. Therefore, we investigated residual T_CIRCM_ and T_RM_ populations in mice that survived a prior B16 challenge (Figure 2A). Relative to age-matched, tumor-inexperienced mice, B16-OVA surviving mice previously treated with isotype antibody exhibited proportional preservation of OT-I T_CIRCM_ across the blood, spleen, and cervical draining lymph nodes (cDLN), as well as OT-I T_RM_ in the IV^–^ brain (Figure 2, B and C). However, tumor-surviving mice previously depleted of OT-I T_CIRCM_ demonstrated not only lasting peripheral depletion, but also a proportional reduction in OT-I brain T_RM_. As OT-I T_RM_ were the only cells capable of protection during B16-OVA challenge in these hosts, we next determined whether numeric perturbations could be discerned in the brain. Here, we observed that OT-I numbers in the brains of tumor-surviving mice varied based on antibody depletion regimen (Figure 2D). Relative to tumor-inexperienced mice, isotype antibody-treated mice exhibited an increase in total OT-I numbers in the brain, whereas a-Thy1.1 antibody-treated mice did not. This result suggests that, in the aftermath of tumorigenic rechallenge, T_CIRCM_ may be called upon and recruited to form new brain T_RM_ via local recall responses. In parallel, a small subset of preexisting brain T_RM_ may fail to persist in tumor-surviving mice, hastening replenishment from the periphery. These findings are supported by prior publications demonstrating the capacity of T_CIRCM_ to form new T_RM_ in tissues upon rechallenge (30, 59). We next aimed to determine whether a core T_RM_ signature was preserved in tumor-surviving mice. Here, the expression of T_RM_-associated markers such as CD69, CD49a, CD103, CXCR6, and PD-1 was broadly preserved among OT-I in the brain irrespective of prior tumor experience, with minimal expression of T_CIRCM_-associated markers such as CD62L (Figure 2, E and F) (15). Notably, tumor-surviving mice exhibited a higher frequency of CD103^+^ OT-I in the IV^–^ brain compared with unchallenged mice, potentially reflecting local antigen encounter in this tissue (Figure 2G). Finally, ex vivo peptide stimulation of OT-I derived from tumor inexperienced and surviving mice revealed a similar ability of brain OT-I to produce IFN-γ across all groups (Figure 2H). In summary, this work suggests that tumor-specific T_RM_ persist in the brain following resolution of a malignant challenge and exhibit preserved functionality.

### Tumor-experienced brain T_RM_ can protect against a second antigen-matched intracranial challenge.

**Figure 3:**
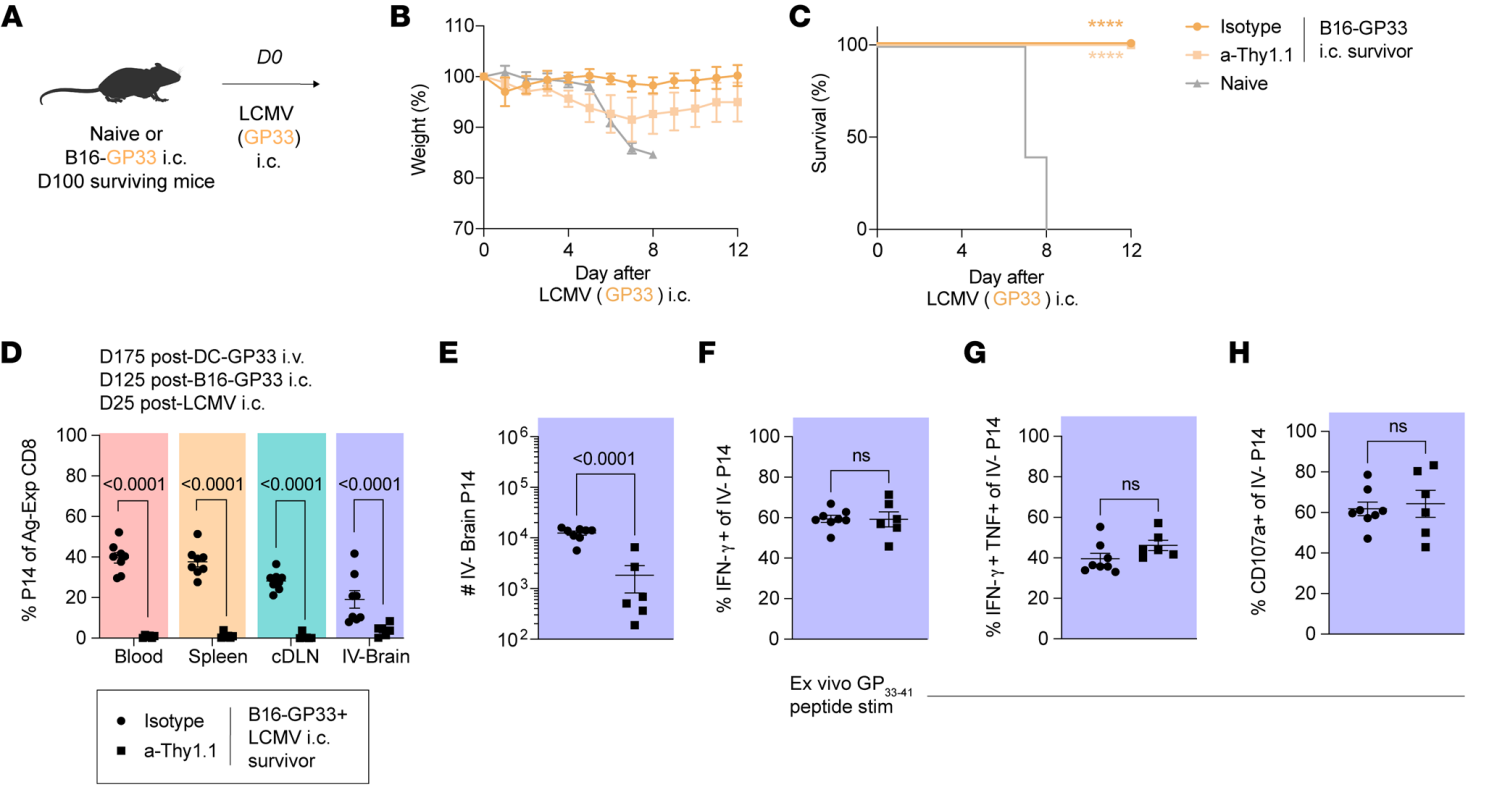
Brain T_RM_ remain functional against a second antigen-matched challenge in tumor-surviving mice. (**A**) Experimental design. At 100 days after B16-GP33 challenge, tumor-surviving mice from Figure 1 or age-matched, tumor naive mice were intracranially (i.c.) injected with LCMV (endogenously expresses the GP_33–41_ epitope). (**B**) Weight loss and (**C**) Kaplan-Meier survival curve of mice injected i.c. with LCMV. (**D**) Frequencies of P14 in the blood, spleen, cDLN, and IV^–^ brain of tumor-surviving isotype and a-Thy1.1 antibody-treated mice. (**E**) Number of P14 in the IV^–^ brain. (**F**) Frequency of IFN-γ^+^, (**G**) IFN-γ^+^TNF^+^, (**H**) CD107a^+^ P14 in the IV^–^ brain following 5–6-hour ex vivo 200 nM GP_33–41_ peptide stimulation. Experiments (**A**–**H**) show concatenated data from 2–3 independent experiments with *n* = 6–10 mice per group total. Statistical significance was determined by log-rank test for survival curves compared with naive mice or Student’s *t* test. Graphs show the mean ± SEM with each symbol representing 1 mouse. Individual *P* values are noted on respective graphs or are otherwise summarized as: ****P < 0.0001. Graphical illustrations were created using BioRender (https://biorender.com).

Metastasis of local or peripheral tumors may occur in temporally distinct waves (54, 60). Therefore, we next investigated the ability of persisting brain T_RM_ in tumor-surviving mice to protect against a second, antigen-matched intracranial challenge. Appreciating that memory immune responses specific for endogenous B16-derived epitopes may be generated during prior tumor elimination, we opted for a rechallenge model system that would solely reengage P14 brain T_RM_ (32). To accomplish this, we inoculated naive and B16-GP33 tumor-surviving mice intracranially with LCMV, representing an expedient rechallenge model system in the brain that offers specificity for the GP_33–41_ epitope recognized by P14 memory T cells (Figure 3A) (27). As expected, naive mice succumbed to intracranial LCMV infection within 8 days of inoculation (Figure 3, B and C). However, B16-GP33 tumor-surviving mice that harbored P14 T_RM_ with or without T_CIRCM_ exhibited minimal weight loss and complete survival against this secondary insult (Figure 3, B and C). We then analyzed P14 T_CIRCM_ populations in blood, spleen, and cervical draining lymph nodes (cDLN), as well as P14 T_RM_ in the brain in B16-GP33 and LCMV surviving mice. Here, we observed outcomes similar to those in Figure 2, where P14 T_CIRCM_ were absent and P14 brain T_RM_ were numerically reduced in a-Thy1.1 antibody treated hosts (Figure 3, D and E). Furthermore, following 2 intracranial insults that normally impart high morbidity and mortality, P14 T_RM_ could still display similar cytokine-producing capacity and cytolysis following ex vivo GP_33–41_ peptide stimulation (Figure 3, F–H, and Supplemental Figure 3, A and B). Taken together, these data indicate that brain T_RM_ remain functionally potent following the resolution of a tumor challenge and may exhibit the capacity to protect against multiple iterations of metastatic seeding in the brain.

### T_RM_ temper brain tumor–associated peripheral immunosuppression and local neuroinflammation.

Metastatic and primary brain tumors dysregulate host immunity in the periphery and brain. Relative to extracranial tumors, intracranial tumors invoke profound lymphoid immunosuppression including lymphoid organ involution, reduced circulating T cell counts, and sequestration of T cells in the bone marrow (2, 3). In parallel, brain tumors produce neuroinflammation and mass effect, necessitating surgical or medical interventions to allay increases in intracranial pressure (61). While peptide-based reactivation of brain T_RM_ elicits transient peripheral immunosuppression and brain inflammation, the longevity, phenotype, and magnitude of these recall responses in response to tumor cell–based challenge remains unclear (29, 62). Here, we examined how antitumor brain T_RM_ moderate immune profiles in the periphery and brain following an intracranial tumor challenge.

**Figure 4:**
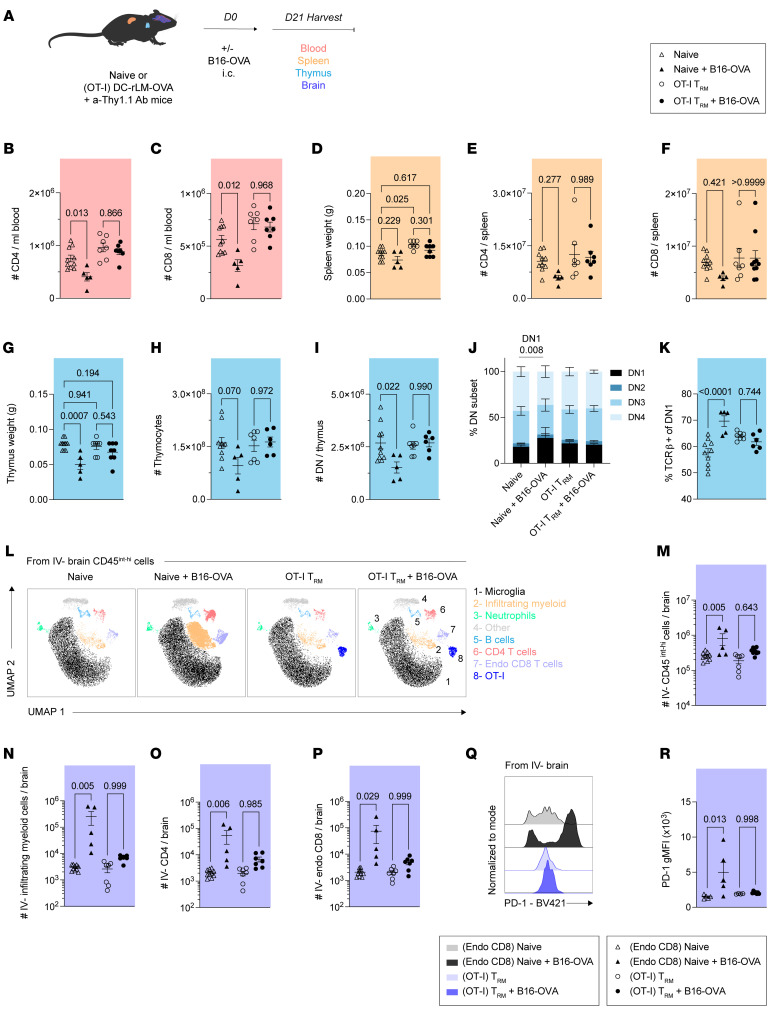
Brain T_RM_ restrain tumor-associated peripheral immunosuppression and local neuroinflammation. (**A**) Experimental design. Naive or (OT-I) DC-rLM-OVA prime-boosted mice previously treated with low-dose a-Thy1.1 depleting antibody were challenged with B16-OVA or no tumor cells. At 21 days after B16-OVA challenge, blood, spleen, thymus, and brain tissues were isolated to determine immune profiles. (**B**) Number of CD4^+^ and (**C**) CD8^+^ T cells in the peripheral blood. (**D**) Splenic weight in grams (g). (**E**) Number of CD4^+^ and (**F**) CD8^+^ T cells in the spleen. (**G**) Thymic weight in grams (g). (**H**) Number of bulk thymocytes and (**I**) double negative (DN) thymocytes. (**J**) Proportion of DN1–4 staging among thymocytes according to CD44 and CD25 expression. (**K**) Frequency of TCR-β^+^ DN1 thymocytes. (**L**) Uniform manifold approximation and projection (UMAP) representations of 36,000 downsampled IV^–^ brain CD45^int-hi^ cells per group concatenated from *n* = 3 representative mice via flow cytometry. (**M**) Number of IV^–^ CD45^int-hi^ cells, (**N**) IV^–^ infiltrating myeloid cells, (**O**) IV^–^ CD4^+^ T cells, and (**P**) IV^–^ endogenous (endo) CD8^+^ T cells. (**Q**) Representative histograms and (**R**) gMFI of PD-1 expression among endo CD8^+^ T cells or OT-I cells according to group. Experiments in (**A**–**P**) show concatenated data from 2 independent experiments with *n* = 5–10 mice per group total. Experiments (**Q** and **R**) show representative data from 1 independent experiment. Statistical significance was determined by 1-way ANOVA with Tukey’s multiple comparison’s test. Graphs show the mean ± SEM with each symbol representing 1 mouse. Individual *P* values are noted on respective graphs. Graphical illustrations were created using BioRender (https://biorender.com).

Naive mice or (OT-I) DC-rLM-OVA prime-boosted mice were treated with a-Thy1.1 antibody to deplete OT-I T_CIRCM_ and were subsequently challenged i.c. with B16-OVA as in Figure 1. At 21 days after tumor cell injection, blood, spleen, thymus, and brain tissues were isolated to comparatively assess immune profiles (Figure 4A). As expected, we observed a reduction in the total number of CD4^+^ and CD8^+^ T cells in the blood of naive, tumor challenged mice, consistent with previous literature (Figure 4, B and C) (2, 3). However, mice harboring antitumor brain T_RM_ did not exhibit peripheral lymphopenia, suggesting an attenuation of this response due to prior immunization (Figure 4, B and C). Spleen profiles revealed trending decreases in splenic weight and CD4^+^/CD8^+^ T cell numbers only in naive tumor-challenged mice, but these differences were not statistically significant at this timepoint (Figure 4, D–F). In the thymus however, we observed a marked reduction in thymic weight in naive, tumor-challenged mice that was not evident in brain T_RM_ harboring hosts (Figure 4G). These mice also exhibited reduced thymocyte cell numbers (Figure 4, H and I). As previously reported, double negative 1 (DN1) thymocyte populations were proportionally increased in naive tumor-bearing hosts with enhanced expression of TCR-β, suggesting impact on thymocyte differentiation (Figure 4, I–K) (2). Within the brain, uniform manifold approximation and projection (UMAP) of IV^–^ CD45^int-hi^ cells revealed diverging representations of infiltrating immune cells (Figure 4L). While naive, tumor-challenged mice exhibited profound increases in peripheral immune cells in the brain, including infiltrating myeloid cells, CD4^+^ T cells, and CD8^+^ T cells, these differences from baseline were not observed among tumor-challenged mice with preexisting tumor-specific brain T_RM_ (Figure 4, M–P). Furthermore, OT-I brain T_RM_ did not exhibit PD-1 upregulation unlike de novo CD8^+^ T cell responses after tumor challenge, expressing PD-1 only to a level expected of T_RM_ populations at homeostasis (Figure 4, Q and R). Altogether, these data indicate that T_RM_-based protection may abrogate the deleterious peripheral immunosuppressive and local neuroinflammatory outcomes affiliated with brain tumors, sustaining host immune equilibrium to more efficiently handle ongoing tumorigenic or pathogenic challenges.

### Peripheral mRNA-LNP vaccination generates antigen-specific brain T_RM_ populations.

Our data thus far have established that peripherally induced brain T_RM_ populations are potent and durable in the face of intracranial malignancy and do not invoke profound neuroinflammation following tumor challenge long term. However, delivery of antigen via autologously loaded, peptide-pulsed DCs and/or *Listeria monocytogenes*–based vectors is unlikely to be meaningfully translated to scale within clinical settings. In contrast, mRNA-LNP vaccine technology has been widely utilized in human populations during the recent SARS-CoV-2 pandemic (63, 64). In mice, mRNA-LNP vaccines exhibit the capacity to generate and rejuvenate T_RM_ populations within tissues such as the lung and liver (44–46, 49). However, the ability of mRNA-LNP vaccines to generate T_RM_ in tissues like the brain after peripheral immunization remains unclear.

**Figure 5:**
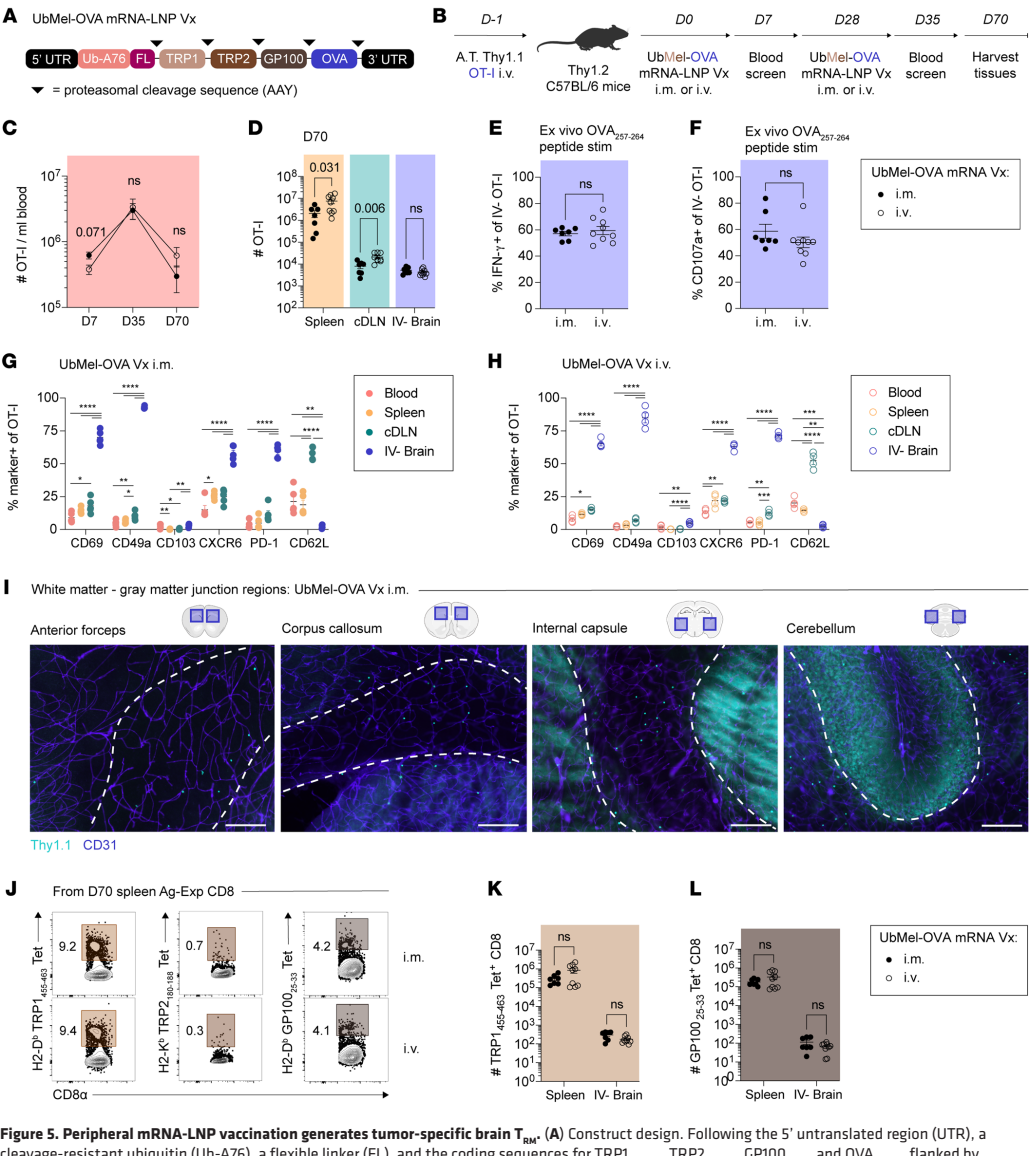
Peripheral mRNA-LNP vaccination generates tumor-specific brain T_RM_. (**A**) Construct design. Following the 5’ untranslated region (UTR), a cleavage-resistant ubiquitin (Ub-A76), a flexible linker (FL), and the coding sequences for TRP1_455–463_, TRP2_180–188_, GP100_25–33_, and OVA_257–264_ flanked by AAY proteasomal cleavage sites were encoded. (**B**) Experimental design. Thy1.2 C57BL/6N mice were adoptively transferred with 10^4^ Thy1.1 OT-I i.v. and immunized one day later with 5 μg UbMel-OVA mRNA-LNP vaccine (Vx) i.m. or i.v. with boosting 28 days later. (**C**) Number of OT-I/ml blood across time. (**D**) Number of OT-I in the spleen, cervical draining lymph nodes (cDLN; number of cells divided by number of cDLN retrieved), and IV^–^ brain. (**E**) Frequency of IFN-γ^+^ and (**F**) CD107a^+^ OT-I in the IV^–^ brain following 5–6-hour ex vivo 1 μM OVA_257–264_ peptide stimulation. (**G**) T_RM_-associated marker expression among OT-I in i.m. and (**H**) i.v. vaccinated hosts. (**I**) Thy1.1^+^ OT-I and CD31^+^ vasculature in white-gray matter junction regions of i.m. vaccinated hosts. Scale bar: 200 μm. (**J**) Representative H2-D^b^ TRP1_455–463_, H2-K^b^ TRP2_180–188_, and H2-D^b^ GP100_25–33_ tetramer (Tet) staining in the spleen. (**K**) Number of TRP1_455–463_- and (**L**) GP100_25–33_-specific CD8^+^ T cells in the spleen and IV^–^ brain. Experiments in (**A**–**F** and **J**–**L**) show concatenated data from 2 independent experiments with *n* = 7–20 mice per group total. Experiments (**G** and **H**) show representative data from 1 of 2 independent experiments with *n* = 4–5 mice per group. Experiments (**I**) show representative images from *n* = 3–4 mice. Statistical significance was determined by Student’s *t* test or 1-way ANOVA with Tukey’s multiple comparison’s test. Graphs show the mean ± SEM with each symbol representing 1 mouse. Individual *P* values are noted on respective graphs or are otherwise summarized as: *P < 0.05, **P < 0.01, ***P < 0.001, ****P < 0.0001. Graphical illustrations were created using BioRender (https://biorender.com).

Herein, we generated an mRNA construct capable of eliciting tumor-specific CD8^+^ T cell responses. Termed UbMel-OVA, this vaccine encodes a string of H2D^b^ and H2K^b^-restricted CD8^+^ T cell epitopes from B16 melanoma, including TRP1_455–463_, TRP2_180–188_, and GP100_25–33_, as well as the model epitope OVA_257–264_ (Figure 5A) (49, 65). This construct was designed with 5′ and 3′ untranslated regions (UTRs) flanking peptide-encoding regions that would be translated upon in vivo administration. To enhance proteasomal degradation of the vaccine protein product and major histocompatibility complex (MHC) loading of resultant peptides, a noncleavable, mutant ubiquitin (Ub-A76) was encoded on the 5′ end, followed by a flexible linker (FL), and the CD8^+^ T cell epitopes of interest. Each epitope was flanked by optimal proteasomal cleavage residues (AAY) (49, 65–67). Purified mRNA constructs were then packaged in LNPs for in vivo administration. We devised a vaccine regimen wherein mice were adoptively transferred with OT-I and prime-boosted 28 days apart via intramuscular (i.m.) or i.v. administration (Figure 5B). Similar expansion and contraction of effector OT-I in the blood was observed in prime-boosted mice irrespective of peripheral delivery route (Figure 5C). At a memory timepoint 70 days after initial immunization, OT-I T_CIRCM_ were increased in i.v. vaccinated hosts (Figure 5D). However, the number of OT-I in the IV^–^ brain did not differ according to vaccination route (Figure 5D). Critically, after ex vivo stimulation, OT-I engendered by either immunization strategy exhibited similar cytokine and cytolytic capacities (Figure 5, E and F). Furthermore, peripheral mRNA-LNP vaccination generated OT-I in the brain exhibited canonical T_RM_ signatures (Figure 5, G and H) (27, 30). These cells distributed widely throughout the brain following peripheral immunization, including near gray matter–white matter junction regions where changes in vascular diameter and blood flow speed generate metastasis-susceptible niches (Figure 5I) (68). We also validated the ability of our mRNA-LNP vaccine to elicit endogenous melanoma-specific T_CIRCM_ and brain T_RM_ responses, with varying efficacy according to specificity (Figure 5, J–L). Collectively, these data illustrate that peripheral mRNA-LNP immunization-based approaches can generate tumor-specific memory CD8^+^ T cells in the periphery and brain.

To identify a suitable, specificity control mRNA-LNP vaccine for tumor challenge studies, we repurposed our previously published mRNA construct for protection against influenza A virus (IAV) and LCMV (49). Termed UbFlu-GP33, this vaccine construct encodes NP_366–374_ and PA_224–233_ epitopes derived from H1N1 Puerto Rico 8 (PR8)-IAV as well as the GP_33–41_ epitope derived from LCMV (Supplemental Figure 4A). To validate the ability of this vaccine to generate bystander memory CD8^+^ T cells, we prime-boosted mice i.m. with the UbFlu-GP33 construct and then queried virus-specific responses in the periphery and brain (Supplemental Figure 4B). Here, we demonstrate expected kinetic expansion and contraction of IAV- and LCMV-specific T_CIRCM_ following vaccination in the blood (Supplemental Figure 4, C–F). We also demonstrate the generation of virus-specific memory CD8^+^ T cells in the brain with T_RM_ phenotypes (Supplemental Figure 4, G–I). Although purposed as a control vaccine in our subsequent tumor challenge studies, we opted to determine whether antiviral brain T_RM_ generated by mRNA vaccination could protect against intracranial infection. Here, we observed that vaccinated mice harboring P14 brain T_RM_ alone protected against an intracranial LCMV challenge (Supplemental Figure 4, J–L). Furthermore, endogenous PA_224–233_-specific brain T_RM_ engendered by vaccination protected against an intracranial rLM-PA_224–233_ challenge (Supplemental Figure 4, M–O). These data not only indicate that mRNA-LNP vaccine-induced bystander responses can be appropriately controlled for in subsequent tumor protection studies, but also hold promise that mRNA-LNP technology can generate pathogen-specific brain T_RM_ that protect against emerging viral encephalitides.

### mRNA-LNP vaccination-induced, tumor-specific T_RM_ durably protect against intracranial malignancy.

**Figure 6:**
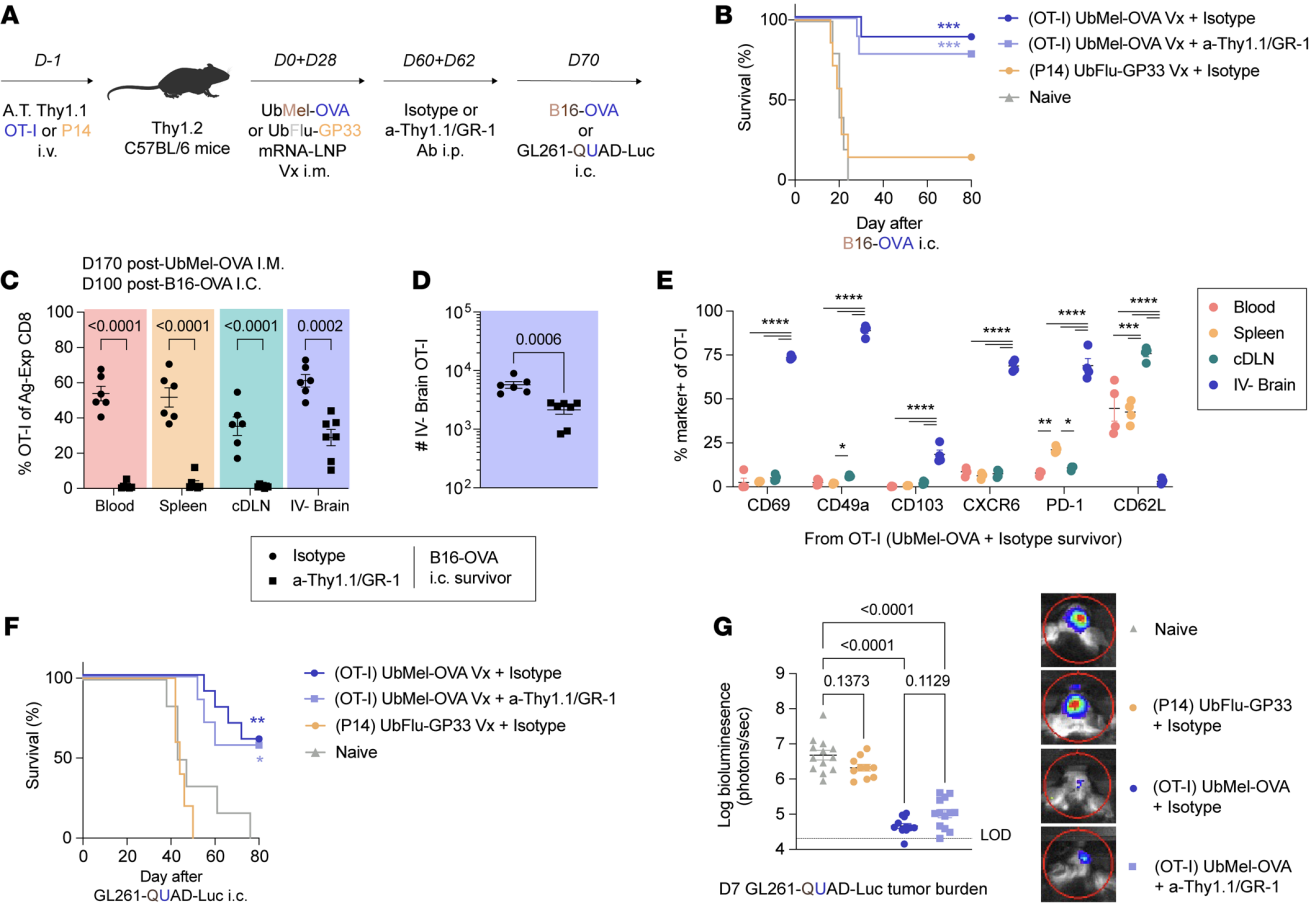
mRNA-LNP vaccination-induced, tumor-specific T_RM_ durably protect against intracranial malignancy. (**A**) Experimental design. Thy1.2 C57BL/6N mice were adoptively transferred with 10^4^ Thy1.1 OT-I or 2 × 10^4^ Thy1.1 P14 i.v. and immunized 1 day later with 5 μg UbMel-OVA or UbFlu-GP33 mRNA-LNP vaccine (Vx) i.m., respectively. Mice were boosted 28 days later ipsilaterally i.m. At 60 and 62 days after initial immunization, mice were treated i.p. with isotype control antibody or a combination of 2 μg a-Thy1.1/ 200 μg a-GR-1 antibodies to deplete TCR-tg and endogenous T_CIRCM_. After 70 days, mice were injected i.c. with B16-OVA cells or glioblastoma GL261-quad cassette-luciferase (GL261-QUAD-Luc) cells. The GL261 cell line expresses 4 model antigens (OVA_257–264_, human GP100_25–33_, OVA_323–339_, Ea_52–68_) and luciferase for bioluminescence imaging. (**B**) Kaplan-Meier survival curves of mice injected with B16-OVA. (**C**) Frequency of OT-I in the blood, spleen, cDLN, and IV^–^ brain of vaccinated B16-OVA tumor surviving isotype- and a-Thy1.1/a-GR-1–treated mice. (**D**) Number of IV^–^ brain OT-I in surviving mice. (**E**) Frequency of T_RM_-associated marker expression among OT-I. (**F**) Kaplan-Meier survival curves of mice injected with GL261-QUAD-Luc i.c. (**G**) Log-transformed bioluminescence signal from GL261-QUAD-Luc–challenged mice at D7 posttumor injection. Experiments (**A**–**D**, **F**, and **G**) show concatenated data from 2–3 independent experiments with *n* = 5–13 mice per group total. Experiments (**E**) show data from 1 of 2 independent experiments with *n* = 4 mice per group. Statistical significance was determined by 1-way ANOVA with Tukey’s multiple comparison’s test or log-rank test for survival curves compared with naive mice. Graphs show the mean ± SEM with each symbol representing 1 mouse. Individual *P* values are noted on respective graphs or are otherwise summarized as: *P < 0.05, **P < 0.01, ***P < 0.001, ****P < 0.0001. Graphical illustrations were created using BioRender (https://biorender.com).

The clinical feasibility of personalized mRNA-LNP vaccination for cancer is emerging rapidly. Therefore, we sought to determine whether mRNA-LNP vaccine-generated brain T_RM_ could offer protection against intracranial tumors. To address this question, we prime-boosted mice i.m. with UbMel-OVA or UbFlu-GP33 mRNA-LNP vaccines. These mice were then either administered isotype or a combination of a-Thy1.1 and a-GR-1 antibodies to robustly deplete TCR-tg and endogenous T_CIRCM_, respectively (Figure 6A and Supplemental Figure 5, A–D). Noting that mice with fulminant brain tumors exhibit an influx of T cells and myeloid cells as seen in Figure 4, we investigated potential off-target effects of the a-GR-1 antibody depletion in eliminating Ly6C^+^ immune cells. While Ly6C^+^ naive CD8^+^ T cell numbers were reduced in the blood of a-GR-1 antibody treated mice, Ly6C^+^ neutrophils and monocytes were fully reconstituted upon time of tumor injection (Supplemental Figure 5, E–I) (69). Mice were then challenged intracranially with B16-OVA or a glioblastoma (GL261) line expressing 4 model epitopes (QUAD), including GP100_25–33_ and OVA_257–264_, as well as luciferase (Luc) to monitor tumor establishment (70). Similar to our peripheral infection-based immunizations, mRNA-LNP vaccination-induced brain T_RM_ durably protected mice against a B16-OVA–based tumor challenge (Figure 6B). Furthermore, we observed numeric and phenotypic preservation of brain T_RM_ long term (Figure 6, C–E). In the context of a GL261 primary brain tumor challenge, where only 2 protective epitopes were conferred by vaccination, we still observed greater protection in vaccination-matched hosts (Figure 6F). We show that UbMel-OVA vaccination substantially lowered tumor burden based on quantitative reduction of brain luminescence as early as 1 week after GL261-QUAD-Luc inoculation (Figure 6G). At this timepoint, tumor-specific, but not bystander, brain T_RM_ robustly proliferated as determined by numeric expansion and Ki-67 expression (Supplemental Figure 6, A–D). Tumor-specific brain T_RM_ also upregulated granzyme B expression (Supplemental Figure 6, E and F). Finally, to validate that protection could be conferred by T_RM_ alone without contributions from the endogenous peripheral T cell compartment, we employed TCR-β KO mice that were adoptively transferred, vaccinated, and depleted of peripheral TCR-tg T cells (Supplemental Figure 7, A and B). Here, vaccine-induced brain P14 T_RM_ still conferred protection against an intracranial B16-GP33 challenge in TCR-β KO mice, validating our antibody depletion approaches (Supplemental Figure 7B). Collectively, this work points to the utilization of anticancer mRNA-LNP vaccines to prevent the establishment or spread of intracranial malignancy.

### Therapeutic mRNA-LNP vaccination generates antigen-specific T cells outside of the tumor microenvironment with T_RM_-like phenotypes and preserved functionality.

Our previous experiments necessitated prophylactic vaccination to establish brain T_RM_ phenotypes prior to challenge. However, mRNA-LNP vaccination may be administered therapeutically (with other concurrent treatments) following the establishment of a diagnosed tumor. While vaccine-induced T cells may undergo programmatic exhaustion in preexisting tumor microenvironments, the ability of mRNA-LNP vaccines to simultaneously engender functional T_CIRCM_ or T_RM_ in distant, nontumor-bearing tissue sites remains undescribed. As metastatic seeding of tumor cells in distant peripheral organs is the primary driver of cancer-related morbidity and mortality, we wished to investigate the ability of functional, tumor-specific T_RM_ cells to be formed in distant tissues following therapeutic mRNA-LNP vaccination (71, 72).

**Figure 7:**
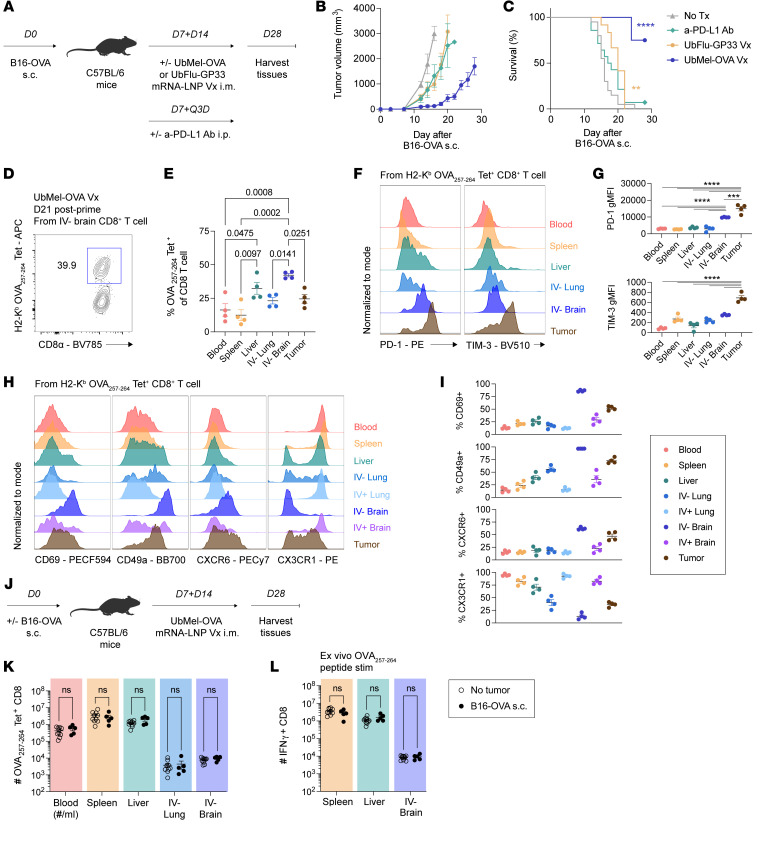
Therapeutic mRNA-LNP vaccination generates tumor-specific CD8^+^ T cells with functional, T_RM_-like phenotypes. (**A**) Experimental design. C57BL/6N mice were injected subcutaneously (s.c.) with B16-F10-OVA. At D7, mice either received 200 μg a-PD-L1 antibody i.p. dosed every 3 days (Q3D) or 5 μg mRNA-LNP vaccine i.m. spaced 7 days apart. (**B**) Tumor growth curves and (**C**) Kaplan-Meier survival curves of mice injected with s.c. B16-OVA tumors. (**D**) Representative gating for H2-K^b^ OVA_257–264_ tetramer (Tet)^+^ CD8^+^ T cells in the IV^–^ brain. (**E**) Frequency of H2-K^b^ OVA_257–264_ Tet^+^ CD8^+^ T cells across tissues. (**F**) Representative histograms and (**G**) gMFI of PD-1 and TIM-3 expression. (**H**) Representative histograms and (**I**) frequency of CD69, CD49a, CXCR6, and CX3CR1 expression. (**J**) Experimental design. C57BL/6N mice were injected s.c. with B16-OVA or no tumor. Mice were then immunized with 5 μg UbMel-OVA i.m. at D7 and D14 after tumor injection with tissue isolation at D28. (**K**) Number of H2-K^b^ OVA_257–264_ Tet^+^ CD8^+^ T cells across tissues in tumor naive and tumor-bearing mice. (**L**) Number of IFN-γ^+^ CD8^+^ T cells across tissues following 5–6 hour ex vivo 1 μM OVA_257–264_ peptide stimulation. Experiments (**C**) show data from 3 concatenated experiments with *n* = 12–20 mice per group total. Experiments (**B** and **D**–**I**) show data from 1 of 3 independent experiments with *n* = 4–5 mice per group. Experiments in (**J**–**L**) show concatenated from 2 independent experiments with *n* = 5–10 mice per group. Statistical significance was determined by Student’s *t* test, 1-way ANOVA with Tukey’s multiple comparison’s test, or log-rank test for survival curves compared with naive mice. Graphs show the mean ± SEM with each symbol representing 1 mouse. Individual *P* values are noted on respective graphs or are otherwise summarized as: ***P < 0.001, ****P < 0.0001. Graphical illustrations were created using BioRender (https://biorender.com).

Here, we leveraged a subcutaneous (s.c.) model of B16-OVA melanoma to establish a peripheral tumor microenvironment. Once tumors became palpable 7 days after injection, mice were either treated with a regimen of a-PD-L1 checkpoint blockade or mRNA-LNP vaccination with monitoring for tumor growth (Figure 7A). Consistent with previous studies employing therapeutic mRNA-LNP vaccination, our UbMel-OVA vaccine outperformed checkpoint blockade and control vaccination against s.c. B16-OVA (Figure 7, B and C) (73–76). We then isolated tumor-specific CD8^+^ T cells following mRNA-LNP vaccination from diverse body compartments. We focused our investigations on common metastatic sites for cutaneous melanoma, including nonlymphoid organs like the liver, lungs, and brain (77, 78). Leveraging tetramer staining, we were able to discern tumor-specific CD8^+^ T cells in the blood, spleen, liver, IV^–^ lung, IV^–^ brain, and tumor with varying representations in mRNA-LNP vaccinated hosts (Figure 7, D and E). Phenotypically, tumor-specific CD8^+^ T cells outside of the tumor did not exhibit profound upregulation of canonical exhaustion markers such as PD-1 and TIM-3 (Figure 7, F and G). Furthermore, the expression of T_RM_-defining markers such as CD69, CD49a, and CXCR6 was pronounced in the IV^–^ brain with some phenotypic acquisition in the liver and IV^–^ lung (Figure 7, H and I). Finally, to determine whether an existing peripheral tumor disrupts mRNA-LNP immunogenicity in distal organs, we vaccinated mice with or without a preexisting peripheral B16-OVA tumor (Figure 7J). Here, we observed that the presence of a peripheral tumor did not impair mRNA-LNP vaccine-induced potency, as lymphoid and nonlymphoid organs harbored similar numbers of tumor-specific CD8^+^ T cells (Figure 7K). Here, a similar number of vaccine-induced CD8^+^ T cells produced IFN-γ in the spleen, liver, and IV^–^ brain following peptide-based stimulation, suggesting retention of T cell function (Figure 7L). In clinical settings, therapeutic mRNA-LNP vaccination has shown promise against solid tumors, with populations of long-lived, nonexhausted, memory-like, neoantigen-specific, blood-surveilling CD8^+^ T cells identified in surviving patients (37, 48). Utilizing mouse models here, our work highlights the added efficacy of mRNA-LNP vaccines in developing protective T_RM_-like CD8^+^ T cells within healthy, but metastasis-susceptible tissues, including the brain.

## Discussion

Here, we have demonstrated the ability of tumor-specific brain T_RM_ to markedly attenuate adverse outcomes in mouse models of intracranial malignancy. Critically, these tumor-specific T_RM_ were generated via translationally relevant, peripheral immunization platforms, including mRNA-LNP vaccine technology. As mRNA-LNP vaccination represents a burgeoning clinical approach to intercept cancer development and spread, our data suggest immunogenic promise even in historically challenging solid organ sites (37, 48).

Despite the global health burden imposed by cancer, medical interventions against tumorigenesis have largely remained therapeutic rather than preventative (35, 47). Vaccination to prevent pathogen-based disease is one of the most successful and cost-effective medical interventions in human history (79). Anticancer vaccines are now being fast tracked by the development of dynamic immunization platforms such as mRNA-LNP technology. mRNA-LNP vaccination offers unique advantages including: (a) the codelivery of multiple tumor antigens that reduce the possibility of immunoediting or antigen loss in tumors, (b) the inclusion of full-length tumor antigens that are not constrained by human leukocyte antigen (HLA) haplotypes, (c) the expediency of diagnostic characterization and design of patient-tailored vaccines, (d) the clinical feasibility of intramuscular injection as an administration route, and (e) the superior immunogenicity compared with viral vector and protein-based vaccination platforms at standard doses (24, 37, 80–82). Simultaneously, genomic sequencing advances are accelerating the identification of neoantigens from primary and metastatic brain tumors that are suitable for vaccine development (39, 50, 83–85). Several therapeutic tumor-targeted mRNA-LNP vaccine clinical trials are also ongoing for primary brain tumors, with early results demonstrating expansion of functional, tumor-specific CD8^+^ T cells and remodeling of the tumor microenvironment in patients with glioblastoma (86–88).

While T_CIRCM_ generated by mRNA-LNP vaccination can be readily sampled from blood in emerging clinical trials, the study of T_RM_ in tissues like the brain will remain challenging unless routinely surveyed via invasive procedures such as lumbar puncture. Therefore, animal models will continue to be critical to understand the optimal mRNA-LNP vaccine (a) design, (b) delivery, and (c) dosing regimens to engender robust T_RM_ populations. Here, our mRNA-LNP vaccine designs enabled the targeted proteasomal cleavage of selected 8–9-mer epitopes derived from sources such as tumors, viruses, and model antigens. However, the ability of full protein-encoding, proinflammatory cytokine-encoding, or self-amplifying mRNA constructs to enhance brain T_RM_ responses is unclear. With respect to delivery, we show that peripheral mRNA-LNP vaccination administered i.m. or i.v. can induce brain T_RM_ populations. Whether these brain T_RM_ outcomes can also be induced via intranasal or topical delivery is unknown. Finally, we utilized a dosing regimen of 5 μg prime-boost vaccinations spaced 1 or 4 weeks apart to numerically enrich brain T_RM_ populations. Whether alternative timing or dosing would enhance T_RM_ generation is untested. Collectively, the design, delivery, and dosing of mRNA-LNP vaccines represents an emerging area of research to enhance brain T_RM_ directed against intracranial tumors and infections.

The study of T cells through the lens of T_RM_ biology is still emerging within the broader fields of neuroimmunology and cancer neuroscience (15). While other tissues that can handle dramatic influxes of immune cells and corresponding mass effect after T_RM_ reanimation, the brain is contained within a fixed skull and will herniate if inflammation exceeds a set volume. Here, we demonstrate that while T_RM_ may proliferate in response to tumor challenge, neuroinflammation is generally tempered. In parallel, unlike other tissues (i.e., skin, lung, intestine) where T_RM_ can migrate to draining lymph nodes and rejoin the circulation during homeostasis or rechallenge, our antibody-based depletion studies do not suggest a conservation of retrograde migration outcomes among brain T_RM_ following a tumorigenic challenge (89). These results may point to an inability of the glymphatic/meningeal lymphatic system to conduct retrograde migration of brain T_RM_ to draining lymph nodes at baseline, prompting further investigation into how brain drainage can be therapeutically enhanced in the context of cancer (1, 90). Future investigations of brain T_RM_ in the context of intracranial malignancy will greatly benefit from the consideration of brain inflammation and glymphatic/meningeal drainage outcomes.

Finally, we show here that virus-specific brain T_RM_ engendered by peripheral mRNA-LNP vaccination protect against intracranial infection. Beyond the obvious clinical utility against emerging neurotropic infections, virus-specific T_RM_ could also be repurposed in brain tumor microenvironments. Whether vaccination-induced, virus-specific T_RM_ generated after tumor establishment could be activated via local administration of antigen-matched oncolytic viruses and/or viral-derived peptides to augment anticancer activities is unknown. Therefore, future investigations of peripheral vaccination combined with local viral-vector based therapeutics could be relevant in the study of brain tumors with low antigenicity.

In summary, this study demonstrates that anti-cancer T_RM_ that are generated in healthy tissue can intercept brain tumorigenesis in mice. Unlike outcomes observed from alternative immunotherapies, brain T_RM_ generated by vaccination are present, potent, and persisting in the face of intracranial malignancy. Ultimately, this work offers hope that T_RM_-based surveillance can thwart cancer development or spread, preventing isolated tumor cells from becoming clinically discernible entities in tissues.

## Methods

### Sex as a biological variable.

Only female mice were used in this study. This decision was made prior to initiation of the studies due to length of experiments with their focus on long-term memory T cells and cancer challenge models that lasted more than 6 months. Fighting between male mice during prolonged housing causes substantial loss of samples, rendering power calculations ineffective and induces potential immune abnormalities in surviving mice due to stress and wounding.

### Mice.

C57BL/6N mice were purchased from the National Cancer Institute. Thy1.1^+^ P14 TCR-tg mice were a gift of Michael Bevan and were bred in-house at the University of Iowa Animal Care Facility. OT-I, Thy1.1^+^, and TCRβ KO mice were obtained from Jackson Laboratories and bred in-house at the University of Iowa Animal Care Facility. Mice used in all experiments were female and 6–10 weeks in age at the onset of experimentation. All animals were handled in accordance with guidelines established by the University of Iowa Institutional Animal Care and Use Committee.

### Adoptive transfer.

Thy1.1^+^ OT-I or P14 TCR-tg CD8^+^ T cells were isolated from naive female donor blood. Red blood cells (RBCs) were lysed using ACK lysis buffer (in-house). Frequencies of TCR-tg cells were determined by flow cytometry. Approximately 10^4^ OT-I cells or 2 × 10^4^ P14 cells were adoptively transferred i.v. via tail vein injection one day prior to infection or immunization.

### Dendritic cell–recombinant Listeria monocytogenes (DC-rLM) prime boost immunization.

Dendritic cell (DC) priming was performed as described previously (51). Briefly, lipopolysaccharide-matured FMS-like tyrosine kinase-3 ligand–induced (Flt-3L–induced) spleen cells were digested with collagenase II/DNase and then incubated for 2 hours at 37°C with 2 μM of peptide (OVA_257–264_, GP_33–41_, or TRP2_180–188_). CD11c^+^ DCs were enriched using anti-CD11c microbeads (Miltenyi Biotec). Subsequently, 5 × 10^5^ peptide-coated DCs were injected i.v. via tail vein injection. Seven days later, mice were boosted with 10^7^ colony forming units (CFU) of attenuated (actA^–/–^, inlB^–/–^), recombinant *Listeria monocytogenes* (rLM) expressing -OVA_257–264_, -GP_33–41_, or -TRP2_180–188_ i.v.

### mRNA vaccine preparation and immunization.

Briefly, an expression cassette was cloned into a pMRNAxp expression plasmid. The expression cassette encoded flanking 5’ and 3’ untranslated regions (UTRs), an A76 mutant ubiquitin (to drive more efficient proteasomal processing), a flexible linker (FL) sequence, and a string of H2-D^b^ and H2-K^b^ restricted optimal 8/9-mer CD8^+^ T cell epitopes specific to the UbMel-OVA vaccine (TRP1_455–463_, TRP2_180–188_, GP100_25–33_, OVA_257–264_) and UbFlu-GP33 vaccine (NP_366–374_, PA_224–233_, GP_33–41_) (49, 65, 67). Each CD8^+^ T cell epitope was flanked by sequences for AAY (alanine, alanine, tyrosine) to provide optimal residues for efficient proteasomal cleavage (65) (Supplemental Table 2). In vitro mRNA expression driven by a type II T7 promoter element, capping, and polyadenylation was achieved using an NEB-ARCA T7 kit (New England Biolabs) supplemented with 50% ψUTP and 5mCTP. RNA was purified using a RNeasy mini kit (Qiagen) according to manufacturer’s instructions. RNA integrity was validated by RNA bleach gel. mRNA was packaged into lipid nanoparticles (LNP) using a Precigenome Nanogenerator Flex microfluidics device. Here, mRNA diluted in 50 mM sodium acetate (pH 5.0) constituted the aqueous phase. The organic phase was prepared by combining a neutral lipid mixture (#PG-SYN_LF1ML, LipidFlex; Precigenome) in 99.5% ethanol with cationic lipid SM-102 at a molar ratio of 6:4. The amine/phosphate (N/P) ratio was fixed at 6 for all LNP preparations. At a 3:1 aqueous:organic flow rate ratio, these fractions were mixed at a total flow rate 3 ml/min using a micromixer chip (#CHP-MIX-4; Precigenome) and a commercial microfluidic mixing system (NanGenerator Flex; Precigenome). The mRNA-LNP was then purified and concentrated using a Milentyi Amicon centrifugation filter with a 10 kD molecular weight cutoff. The encapsulation efficiency and encapsulated mRNA concentration was confirmed using a Quant-iTTM Ribogreen Assay Kit (Thermo Fisher). LNP size was measured at 90–125 nm using a Zetasizer Nano ZS. Mice were vaccinated i.m. in the right quadriceps or i.v. via tail vein injection with 2.5 μg (Supplemental Figure 7) or 5 μg (all other figures) of encapsulated mRNA and were boosted 7 or 28 days later in the same manner.

### Antibody depletion and blockade.

To deplete Thy1.1^+^ OT-I or P14 T_CIRCM_, mice received 1 dose of 2 μg a-Thy1.1 antibody i.p. (clone 19E12; BioXcell) (27). To deplete endogenous T_CIRCM_, mice received 2 doses of 200 μg a-GR-1 antibody i.p. (clone NIMP-R14; BioXcell) spaced 2 days apart (58). To block PD-L1 signaling, mice received serial doses of 200 μg a-PD-L1 antibody i.p. (clone 10F.9G2; BioXcell) spaced 3 days apart (32).

### Tumor cell lines and injections.

The murine B16-F10, B16-OVA, and B16-GP33 melanoma cell lines were grown in RPMI 1640 media (Gibco) with L-glutamine supplemented with 10% fetal calf serum (FCS) and supplementum complementum (made in-house). Confluent cells were detached with 0.25% Trypsin-EDTA (Gibco). Mice were injected with 10^2^ B16 cells in a total volume of 10 μl i.c. Mice were alternatively injected with 2 × 10^5^ B16 cells in the right flank in a total volume of 100 μl s.c.

The murine GL261-quad cassette-luciferase (GL261-QUAD-Luc) glioblastoma cell line was kindly provided by Aaron Johnson by way of John Ohlfest (Masonic Cancer Center, University of Minnesota, Minneapolis, Minnesota). This quad cassette cell line is engineered to express 4 model antigens (OVA_257–264_, human GP100_25–33_, OVA_323–339_, and the alloantigen Ea_52–68_) as well as luciferase (Luc) to enable bioluminescence imaging. GL261-QUAD-Luc cells were grown in Dulbecco’s modified Eagle media (DMEM, Gibco) with L-glutamine supplemented with 10% FCS and supplementum complementum (made in-house). Confluent cells were detached with TrypLE Express Enzyme (Gibco). Mice were injected with 2 × 10^4^ GL261 cells in a total volume of 2 μl i.c. containing 2.5% methylcellulose.

### Lymphocytic choriomeningitis virus intracranial infection.

Lymphocytic choriomeningitis virus (LCMV) infection was performed with 10^3^ plaque forming units (PFU) of LCMV strain Armstrong in a total volume of 10 μl i.c.

### Attenuated recombinant rLM intracranial infection and bacterial counts.

rLM infection was performed with 10^2^ CFU of attenuated (actA^–/–^, inlB^–/–^), rLM expressing -PA_224–233_ in a total volume 10 μl i.c. To determine rLM bacterial burden, brains were isolated and weighed in 5 ml 0.2% Igepal (Millipore Sigma). The tissue was homogenized and plated with 10-fold serial dilutions on Tryptic Soy Broth (TSB) / streptomycin plates. Colony counts were determined after overnight plate incubation at 37°C.

### IVIS.

GL261-QUAD-Luc burden was assessed by IVIS at specified timepoints posttumor injections. Approximately 5–10 minutes prior to IVIS imaging, mice were injected i.p. with 3 μg of VivoGlo D-Luciferin (ProMega) and were anesthetized with 2% isoflurane during image acquisition. Luminescence in equal-sized areas of interest were quantified using Xenogen Living Image software (Caliper Life Sciences).

### Tumor measurements.

Tumor volumes were monitored every other day using a vernier caliper. Tumor volume was calculated using the equation: V = (4 × 3.14 × A × B^2^)/3, where V is volume (mm^3^), A is the largest diameter (mm), and B is the smallest diameter (mm) (73).

### Tissue collection and cellular isolation.

Mice received an i.v. injection of 2 μg anti-CD45 (30-F11, BioLegend) conjugated to a fluorophore 3 minutes prior to tissue harvest. Blood samples were collected via retroorbital bleed. RBCs were lysed with ACK lysis buffer. Spleens were isolated and dissociated through a 70 μm filter followed by RBC lysis with ACK lysis buffer. Pooled (4–8) cervical draining lymph nodes, thymi, and subcutaneous B16 tumors were isolated and dissociated through a 70 μm filter. Livers were isolated and digested in liver digestion buffer (Gibco) for 1 hour at 37°C. Lungs and kidneys were isolated and digested in Collagenase II/DNase (Millipore Sigma) for 1 hour at 37°C. Livers, lungs, and kidneys were dissociated through a 70 μm filter and separated using a 35% Percoll (GE Healthcare) gradient spun at 510 × *g* for 10 minutes at 25°C followed by RBC lysis with Vitalyse (CMDG). Brain and meninges were isolated and digested in CollagenaseD/DNase (Millipore Sigma) for 45 minutes at 37°C. Brain and meninges were then dissociated through a 70 μm filter. Brains were further separated using a layered 70% and 37% Percoll (GE Healthcare) gradient spun at 910 × *g* for 20 minutes at 25°C. Brain mononuclear cells were collected at the gradient interface. Following meningeal extraction, cleaned cranial bone was mashed with a mortar and pestle to extract bone marrow. Cranial bone samples were filtered through a 70 μm filter followed by RBC lysis with Vitalyse (CMDG).

### Cell staining and flow cytometry.

To assess antigen-specific CD8^+^ T cell populations, in-house prepared H2-D^b^ or H2-K^b^ restricted tetramers specific for OVA_257–264_, TRP1455_–463_, TRP2_180–188_, GP100_25–33_, NP_366–374_, and PA_224–233_ peptides were incubated with cells at a dilution of 1:100 with Fc block at 4°C prior to surface antibody staining. Otherwise, single-cell suspensions were surface stained with Fc block for 30 minutes at 4°C with a panel of fluorescently labeled antibodies (Supplemental Table 1). Samples were then fixed with Cytofix (BD Bioscience) at 25°C for 10 minutes. To assess cytokine production, single-cell suspensions were incubated in the absence or presence of 1 μM OVA_257–264_ peptide or 200 nM GP_33–41_ peptide with brefeldin A (BioLegend) for 5–6 hours at 37°C. Stimulated and unstimulated cells were similarly stained for surface markers at 4°C, permeabilized using a FoxP3 transcription factor staining kit (Tonbo/Cytek), and stained for cytokines at 25°C. After staining, cells were transferred to 1.2 ml microtiter tubes (Thermo Fisher) in FACS buffer and approximately 15 μl of Count Bright Absolute Counting Beads were added to each tube (Thermo Fisher). Flow cytometry data were acquired on a LSRFortessa flow cytometer (BD Bioscience) and analyzed using FlowJo software v.10 (FlowJo LLC) using Downsample and UMAP plug-ins. Cells were analyzed utilizing the gating strategies available in Supplemental Figure 8 and Supplemental Figure 9.

### Brain immunohistochemistry.

Freshly extracted whole brain specimens were embedded within Peel-A-Way embedding molds (Thermo Fisher) containing 37°C liquid 4% low melting-point agarose (Promega) dissolved in 1X PBS. Molds were cooled at 4°C until the agarose mixture solidified. The embedded molds were then placed on a PELCO easiSlicer vibratome (Ted Pella) in a cranial-to-caudal position vertically. Sections of 200 μm were cut spanning from olfactory bulb to brainstem and were placed in wells containing cold 1X PBS. Brain tissue sections were stained with antibodies for 2 hours at 4°C in 1X PBS with Fc block (Supplemental Table 1). Stained brain tissue was washed twice in PBS and mounted on Superfrost Plus microscope slides (Thermo Fisher) to dry. Slides were coverslipped in ProLong Gold Antifade Mountant containing DAPI (Thermo Fisher). Whole-slide images were acquired using an Olympus VS120 slide-scanning microscope and reviewed in OlyVIA software v.2.9.1 (Olympus). ImageJ v.1.53t and Adobe Photoshop v.21.0.3 were utilized for image processing. Neuroanatomical localization was cross-referenced with the Allen Brain Atlas (http://www.brain-map.org).

### Statistics.

All statistical analyses were performed using GraphPad Prism (v9.0). When indicated, 2-tailed unpaired Student’s *t* tests were performed when comparing 2 independent groups, paired *t* tests when comparing 2 paired groups, and 1-way ANOVA with Tukey’s multiple comparisons test when comparing more than 2 groups. Significant differences in survival were determined using a log-rank (Mantel-Cox) test. *P* values are indicated in individual figures, and *P* < 0.05 was considered statistically significant.

### Study approval.

Mouse studies were performed at the University of Iowa with approval from the Institutional Animal Care and Use Committee under protocol 3051102.

### Data availability.

All raw data associated with the main article and supplemental material are included in the Supporting Data Values file. Other data are available upon request.

## Author contributions

MRM, CMS, MH, VPB, and JTH designed the experiments. MRM, CMS, MH, SKK, LLP, SCH, RH, CEF, MH, LSH, SAA, and TGB performed the experiments. RH, FJ, AJJ, CSC, NSB, and AKS provided reagents, methods, equipment and/or mice for some experiments. MRM, CMS, and MH analyzed data from the experiments. MRM, CMS, and JTH wrote the manuscript with feedback from all authors. 

## Conflict of interest

SCH and CSC are co-founding members of Geminii, Inc. and are inventors on patents (AU2019223992B2, US11071875B2, and US11850440B2) licensed to Geminii, Inc., both of which are unrelated to experiments performed and data collected in this study.

## Funding support

This work is the result of National Institutes of Health (NIH) funding, in whole or in part, and is subject to the NIH Public Access Policy. Through acceptance of this federal funding, the NIH has been given a right to make the work publicly available in PubMed Central. This work was made possible through the following grants including:

- NIH R01AI167847 (JTH).
- NIH R21AI178159 (JTH).
- NIH R01AI114543 (JTH, VPB).
- NIH R35GM134880 (VPB).
- NIH R01NS122174 (AJJ).
- NIH R01NS103212 (AJJ).
- NIH R01AI125446 (NSB).
- NIH R01AI127481 (NSB).
- NIH R01AI167058 (NSB).
- NIH T32AI007260 (MH, CEF).
- NIH T32CA078586 (SCH).
- NIH F32AI174382 (MH).
- NIH K99AI190129 (MH).
- NIH T32GM139776 (MRM, SAA).
- University of Iowa Graduate College Post-Comprehensive Research Fellowship (MRM, SKK).
- University of Iowa Ballard and Seashore Fellowship (MRM).
- Howard Hughes Medical Institute Hanna H. Gray Fellows Program (CEF).

## Supplementary Material

Supplemental data

Supporting data values

## Version 1 04/15/2026

Electronic publication
