# Supplementary material for: Peripheral vaccination-induced brain-resident memory CD8+ T cells durably protect mice against intracranial malignancy
Source: J Clin Invest. 2026 Apr 15;136(8):e197812. doi: 10.1172/JCI197812 (PMC13078870; doi:10.1172/JCI197812)
Supplement: Supplemental data [file jci-136-197812-s009.pdf]

Supplemental Figures.

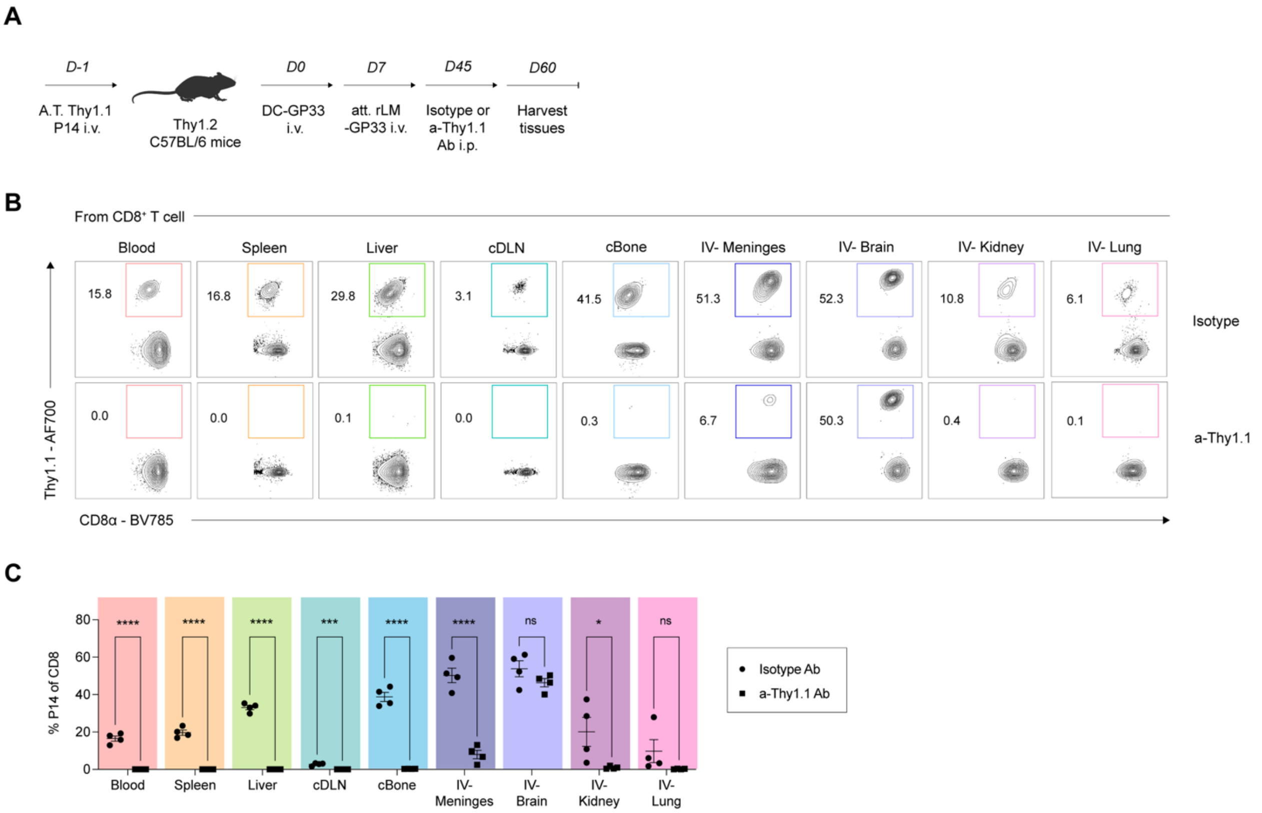

Supplemental Figure 1. a-Thy1.1 antibody treatment systemically depletes TCR-tg T cells while preserving brain T_{RM}. (**A**) Experimental design. Thy1.2 C57BL/6N mice were adoptively transferred with $2x10^4$ Thy1.1 P14 intravenously (i.v.) one day prior to i.v. injection with GP_{33-41} peptide-pulsed, LPS-matured dendritic cells (DC-GP33). After DC prime, mice were boosted with attenuated recombinant *Listeria monocytogenes* expressing GP_{33-41} (att. rLM-GP33). At a memory timepoint 45 days after DC prime, mice were treated with 2 μg of isotype control or a-Thy1.1 antibody intraperitoneally (i.p.) to deplete P14 memory T cells in select tissues. Approximately 3 minutes prior to tissue harvest on D60, mice were injected intravenously with a fluorophore conjugated anti-CD45 antibody to distinguish P14 T cells in the vasculature (IV+) versus P14 T cells localized in tissues (IV-) at the time of isolation. (**B**) Representative flow plots of $Thy1.1^+$ $CD8^+$ T cells in the blood, spleen, liver, cervical draining lymph nodes (cDLN), cranial bone (cBone), IV- meninges, IV- brain, IV- kidney, and IV- lung following isotype or a-Thy1.1 antibody treatment. (**C**) Frequency of $Thy1.1^+$ P14 T cells in respective tissues following antibody treatments. Experiments in (**B-C**) show representative data from 1 independent experiment with n=4 mice per group. Statistical significance was determined by student's t-test. Graphs show the mean ± s.e.m. with each symbol representing one mouse. Individual *p* values are summarized as: $*p < 0.05$, $**p < 0.01$, $***p < 0.001$, $****p < 0.0001$. Graphical illustrations were created using BioRender (https://biorender.com).

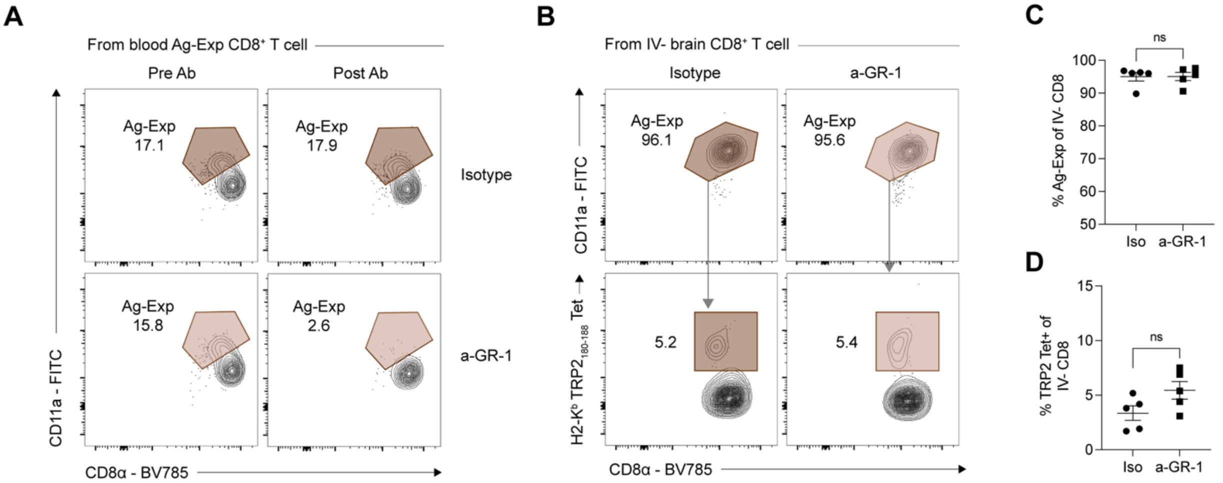

Supplemental Figure 2. a-GR-1 antibody treatment effectively depletes T_{CIRCM} while preserving brain T_{RM}. (**A**) Representative flow plot of $CD11a^{hi}$ Ag-Exp $CD8^+$ T cells in the blood of DC-rLM-TRP2 prime-boosted mice pre- and post- antibody treatment with isotype control or a-GR-1 antibodies. (**B**) Representative flow plot of Ag-Exp $CD8^+$ T cells and $TRP2_{180\text{-}188}$-specific $CD8^+$ T cells in the IV- brain from DC-rLM-TRP2 prime-boosted mice 10 days following isotype or a-GR-1 antibody treatment. (**C**) Frequency of Ag-Exp $CD8^+$ T cells and (**D**) $TRP2_{180\text{-}188}$-specific $CD8^+$ T cells in the IV- brain after antibody treatments. Experiments in (**A**) show representative data from 2 independent experiments with n=7-9 mice per group total. Experiments in (**B-D**) show representative data from 1 of 2 independent experiments with n=5 mice per group. Statistical significance was determined by student's t-test. Graphs show the mean ± s.e.m. with each symbol representing one mouse.

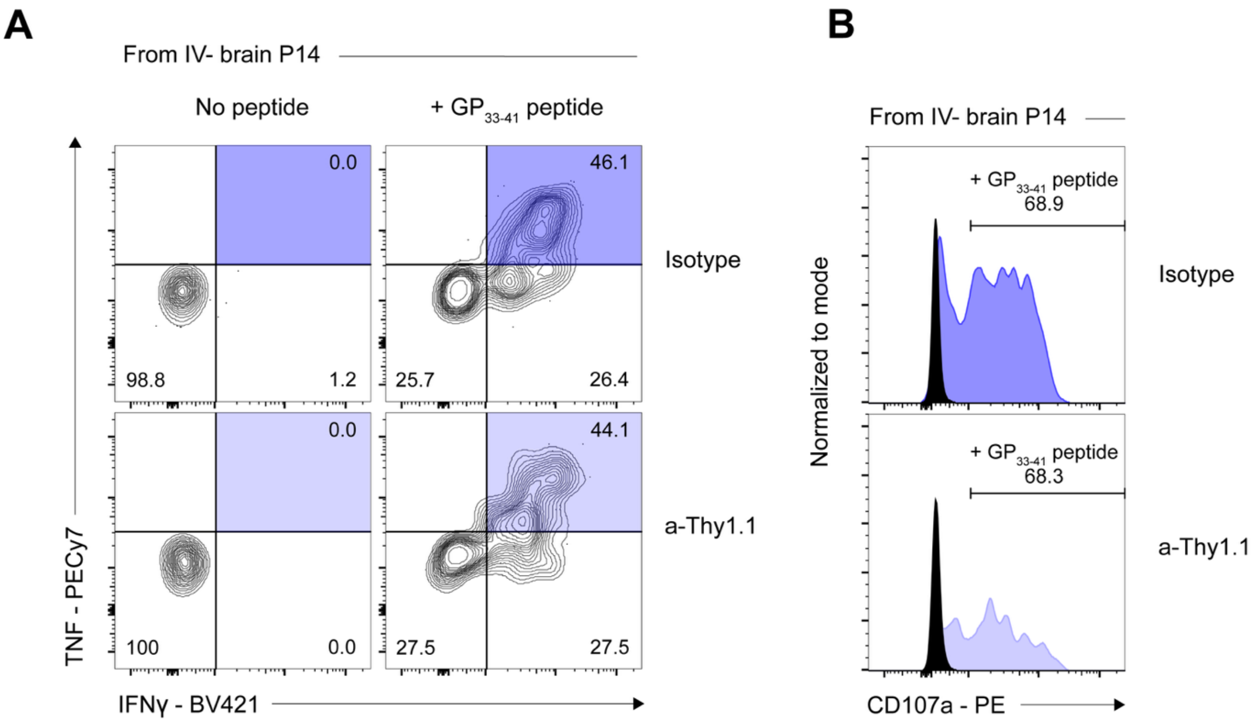

Supplemental Figure 3. Representative gating of cytokine production and cytolytic capacity of brain T_{RM} following ex vivo GP_{33-41} peptide stimulation. (**A**) Expression of IFN-γ and TNF among IV- brain P14 following 5-6-hour ex vivo 200 nM GP_{33-41} peptide stimulation or no peptide control in B16-GP33 and LCMV i.c. surviving mice previously treated with isotype or a-Thy1.1 antibodies. (**B**) Expression of CD107a among IV- brain P14 following peptide stimulation or no peptide control (black histograms).

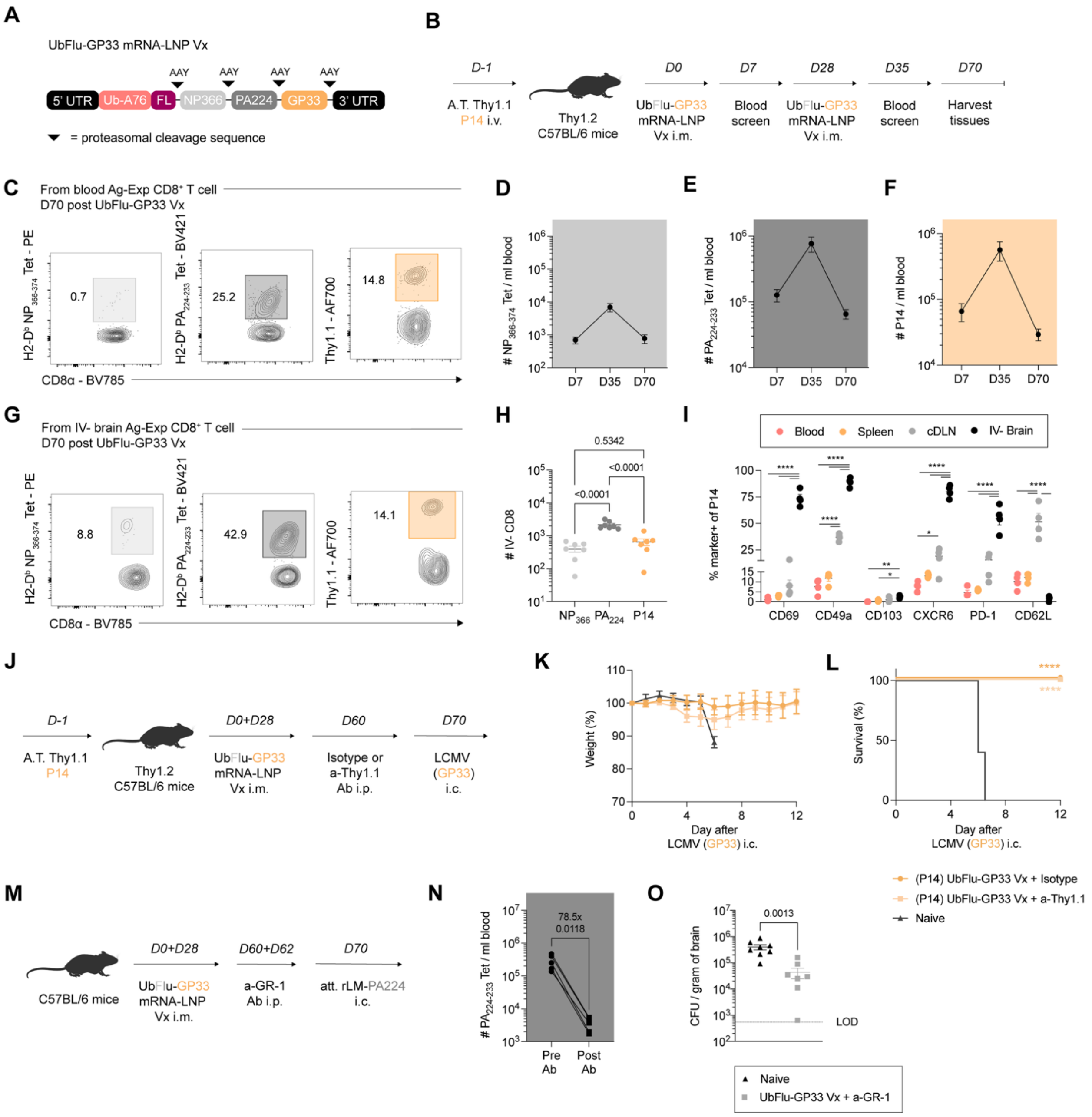

Supplemental Figure 4. Peripheral mRNA-LNP vaccination generates protective virus-specific brain T_{RM}. (**A**) Construct design. Same as in Figure 5 but with $NP_{366-374}$, $PA_{224-233}$, and GP_{33-41} coding sequences. (**B**) Thy1.2 C57BL/6N mice were adoptively transferred with $2x10^4$ Thy1.1 P14 i.v. and immunized one day later with 5 µg UbFlu-GP33 mRNA-LNP vaccine (Vx) i.m. with boosting 28 days later. (**C**) Tetramer and Thy1.1$^+$ P14 virus-specific CD8$^+$ T cell staining. (**D-F**) Number of virus-specific CD8$^+$ T cells in blood across time. (**G**) Representative staining and (**H**) numbers of virus-specific CD8$^+$ T cells in the IV- brain. (**I**) T_{RM}-associated marker expression among P14. (**J**) Mice were vaccinated as in (B), treated i.p. with isotype or anti-Thy1.1 antibody (Ab) to deplete P14 T_{CIRCM}, and challenged i.c. with LCMV. (**K**) Weight loss and (**L**) survival after LCMV i.c. challenge. (**M**) C57BL/6N mice were immunized, treated with a-GR-1 Ab i.p. to deplete T_{CIRCM}, and challenged i.c. with att. rLM-$PA_{224-233}$. (**N**) Number of H2-D^b $PA_{224-233}$ Tet$^+$ CD8$^+$ T cells / ml of blood. (**O**) rLM-$PA_{224-233}$ colony forming units (CFU) / gram of

brain tissue with limit of detection (LOD) noted. Experiments in (**A-H; O**) show concatenated data from 2 independent experiments with n=7-15 mice per group total. Experiments in (**K-L**) show concatenated data from 3 independent experiments with n=7-10 mice per group total. Experiments in (**I; N**) show data from 1 of 2 independent experiments with n=4-5 mice. Statistical significance was determined by Student's t-test, paired t-test, one-way ANOVA with Tukey's multiple comparison's test, or log-rank test for survival curves compared to naïve mice. Graphs show the mean ± s.e.m. with each symbol representing one mouse. Individual *p* values are noted on respective graphs or are otherwise summarized as: **p* < 0.05, ***p* < 0.01, ****p* < 0.001, *****p* < 0.0001. Graphical illustrations were created using BioRender (https://biorender.com).

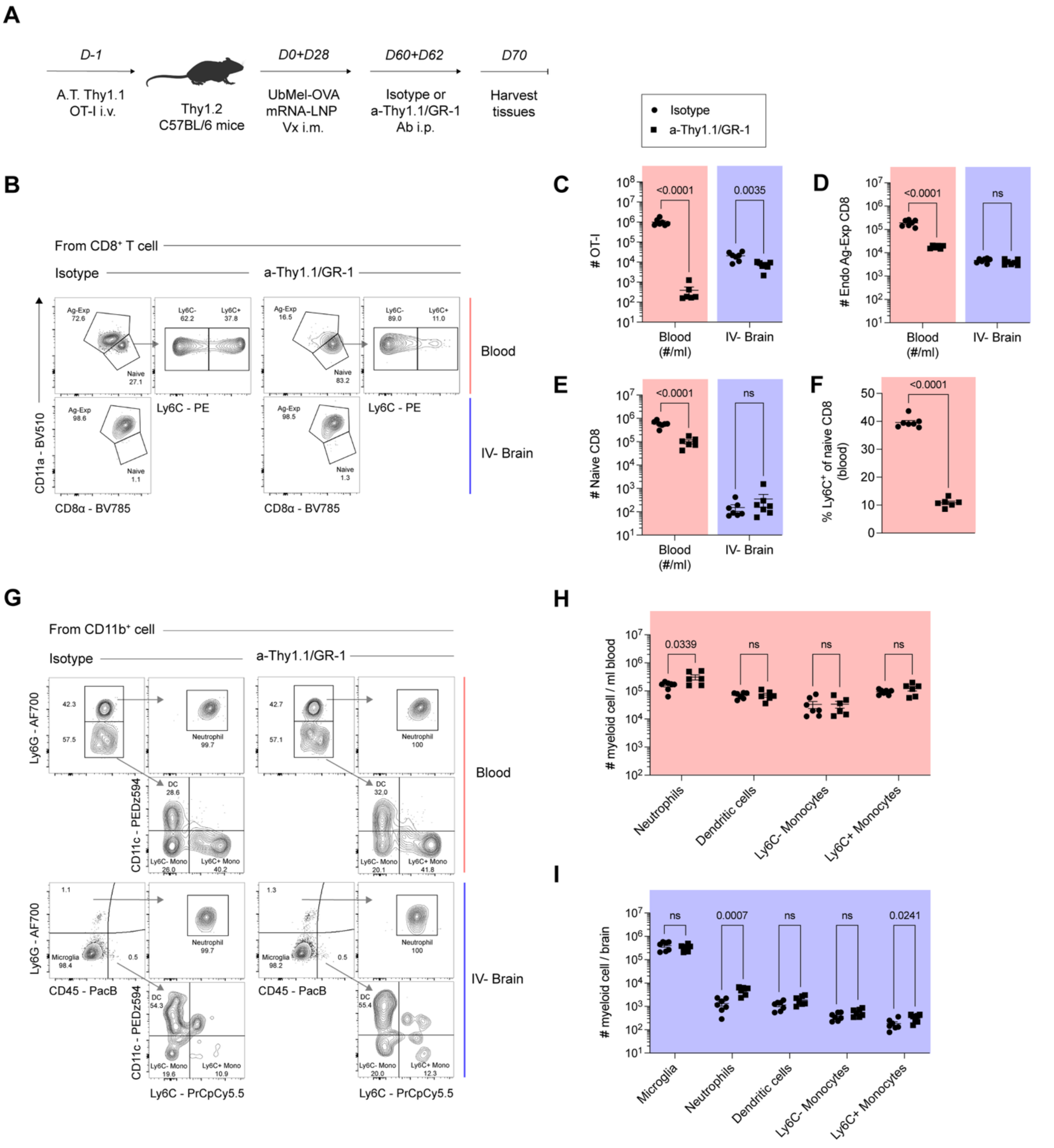

Supplemental Figure 5. Combination a-Thy1.1/GR-1 antibody depletion impact on CD8⁺ T cell and myeloid cell compartments. (**A**) Experimental design. Thy1.2 C57BL/6N mice were adoptively transferred with 10^4 Thy1.1 OT-I i.v. and immunized one day later with 5 μg UbMel-OVA mRNA-LNP vaccine (Vx) i.m. Mice were boosted 28 days later with 5 μg UbMel-OVA mRNA-LNP vaccine ipsilaterally i.m. At 60 and 62 days after initial immunization, mice were treated i.p. with isotype control antibody or a combination of 2 μg a-Thy1.1 / 200 μg a-GR-1 antibodies to broadly deplete TCR-tg and endogenous T_{CIRCM}. After 70 days, tissues were harvested. (**B**) Representative gating of antigen-experienced (Ag-Exp) and naïve $CD8^+$ T cells in the blood and IV- brain following antibody depletion. (**C**) Number of OT-I, (**D**) endogenous Ag-Exp $CD8^+$ T cells, and (**E**) naïve $CD8^+$ T cells in the blood and IV- brain of antibody treated

hosts. (**F**) Frequency of Ly6C$^+$ naïve CD8$^+$ T cells in the blood. (**G**) Representative gating of CD11b$^+$ myeloid cells in the blood and IV- brain of antibody treated hosts. (**H**) Number of neutrophils, dendritic cells, Ly6C- monocytes, and Ly6C$^+$ monocytes in the blood of antibody treated hosts. (**I**) Same as (H) but for IV- brain samples encompassing microglia populations. Experiments in (**A-I**) show concatenated data from 2 independent experiments with n=7-8 mice per group total dependent on tissue type. Statistical significance was determined by Student's t-test. Graphs show the mean ± s.e.m. with each symbol representing one mouse. Individual *p* values are noted on respective graphs. Graphical illustrations were created using BioRender (https://biorender.com).

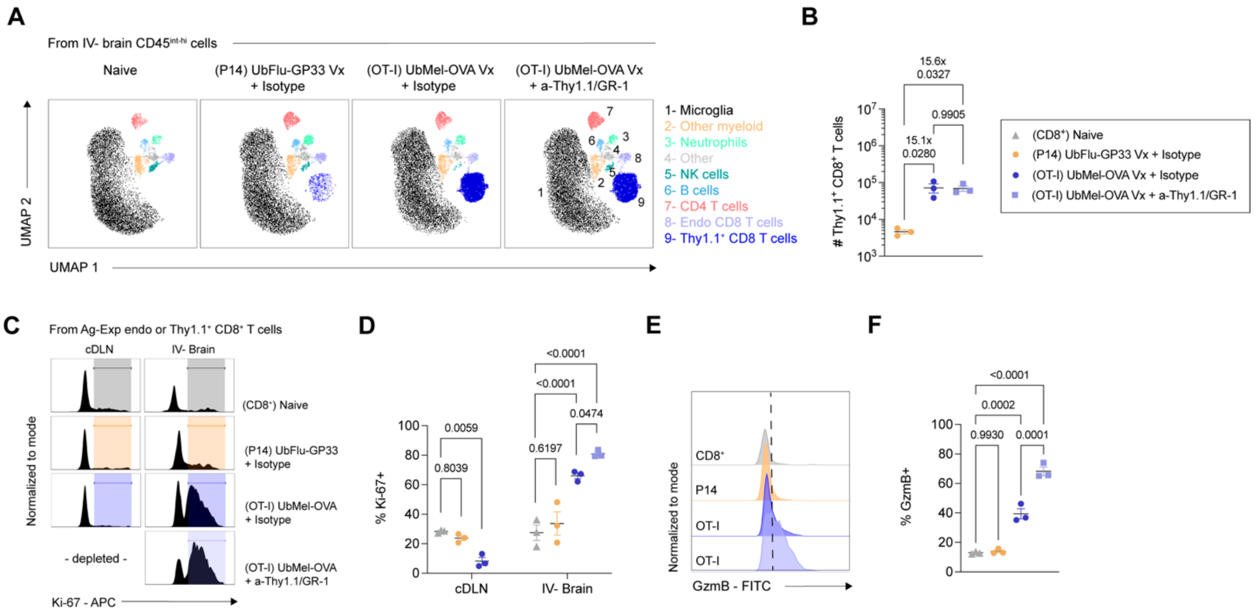

Supplemental Figure 6. Tumor-specific brain T_{RM} exhibit enhanced proliferative and cytolytic capacities acutely after tumor challenge. (**A**) Uniform manifold approximation and projection (UMAP) representations of 45,000 downsampled IV- brain CD45^{int-hi} cells per group concatenated from n=3 representative mice via flow cytometry 7 days after GL261-QUAD-Luc i.c. challenge. (**B**) Number of IV- Thy1.1$^+$ CD8$^+$ T cells in the brains of UbFlu-GP33 or UbMel-OVA vaccinated mice. (**C**) Representative histograms and (**D**) gMFI of Ki-67 expression among endogenous CD8$^+$ or Thy1.1$^+$ CD8$^+$ T cells in the cDLN or IV- brain of naïve or vaccinated mice. (**E**) Representative histograms and (**F**) gMFI of granzyme B (GzmB) expression among CD8$^+$ or Thy1.1$^+$ CD8$^+$ T cells in the IV- brain of naïve or vaccinated mice. Experiments in (**A-F**) show data from 1 independent experiment. Statistical significance was determined by one-way ANOVA with Tukey's multiple comparison's test. Graphs show the mean ± s.e.m. with each symbol representing one mouse. Individual *p* values are noted on respective graphs.

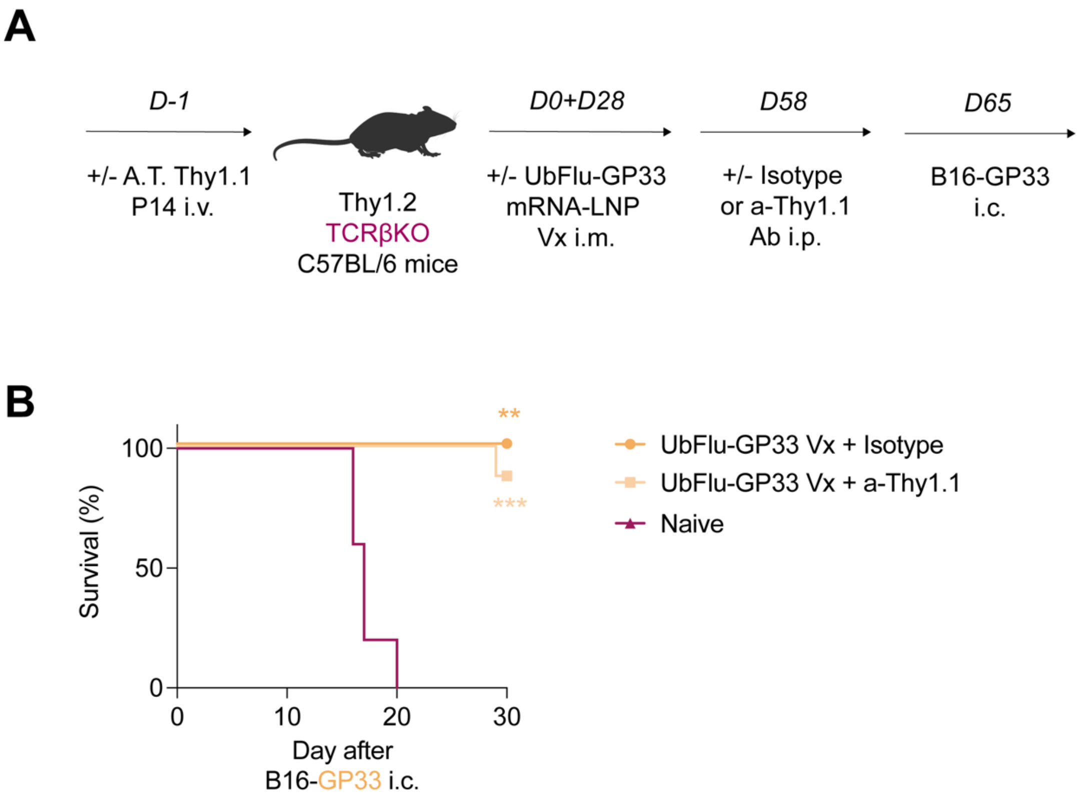

Supplemental Figure 7. T_{RM}-mediated protection against intracranial malignancy occurs independently of a peripheral T cell compartment. (**A**) Experimental design. Thy1.2 TCRβKO C57BL/6N mice were adoptively transferred with $2x10^4$ Thy1.1 P14 i.v. and immunized one day later with 2.5 μg UbFlu-GP33 mRNA-LNP vaccine i.m. Mice were ipsilaterally boosted 28 days later. At 58 days after initial immunization, mice were treated i.p. with 2 μg of isotype control or a-Thy1.1 antibody to deplete P14 T_{CIRCM}. After 65 days, mice were injected i.c. with B16-GP33 cells. (**B**) Kaplan-Meier survival curves of mice injected with B16-GP33 i.c. Experiments in (**B**) show data from 1 of 2 independent experiments with n=5-8 mice. Statistical significance was determined by log-rank test for survival curves compared to naïve mice. Individual *p* values are summarized as: $*p < 0.05$, $**p < 0.01$, $***p < 0.001$, $****p < 0.0001$. Graphical illustrations were created using BioRender (https://biorender.com).

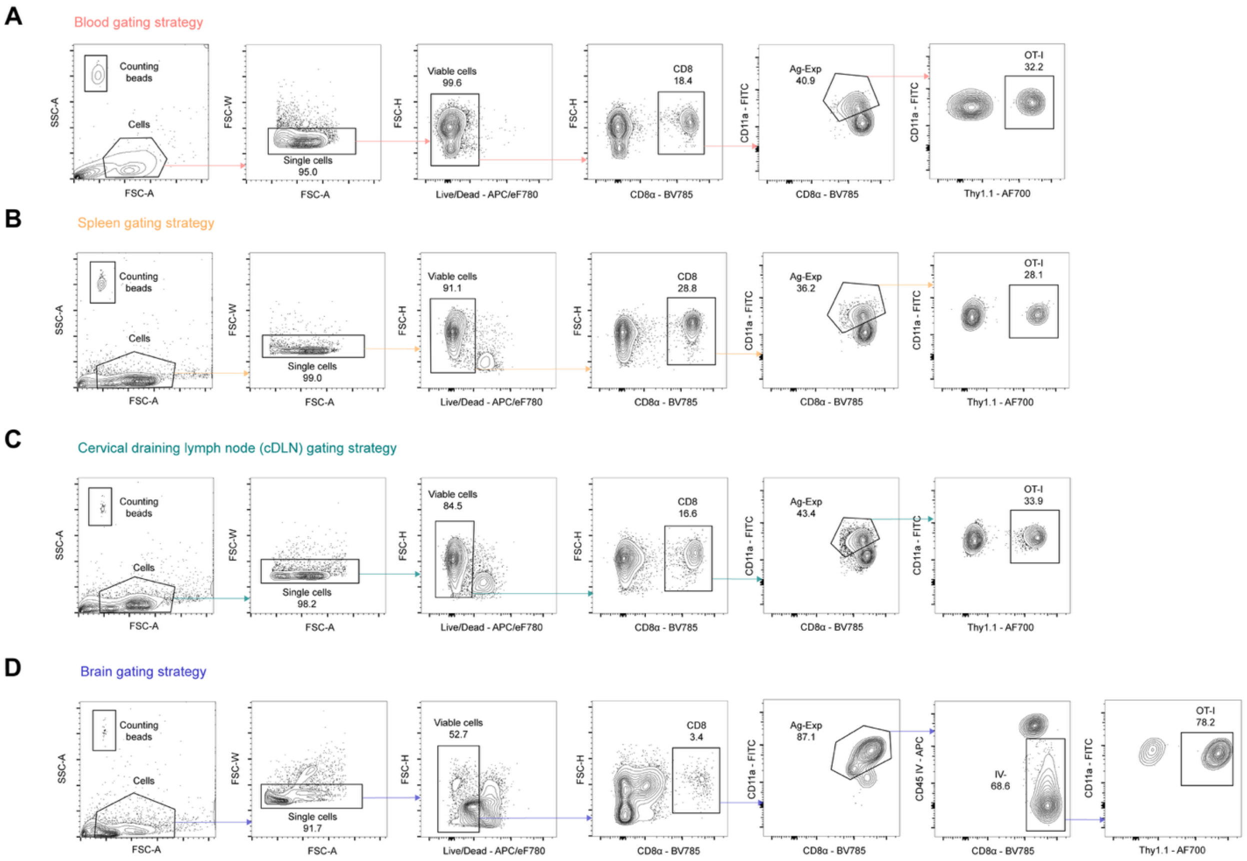

Supplemental Figure 8. Representative gating strategy for CD8$^+$ T cell populations in tissues. (**A**) Representative gating strategy for Ag-Exp CD8$^+$ T cells and/or TCR-tg CD8$^+$ T cells in the blood, (**B**) spleen, (**C**) cervical draining lymph nodes, and (**D**) IV- brain.

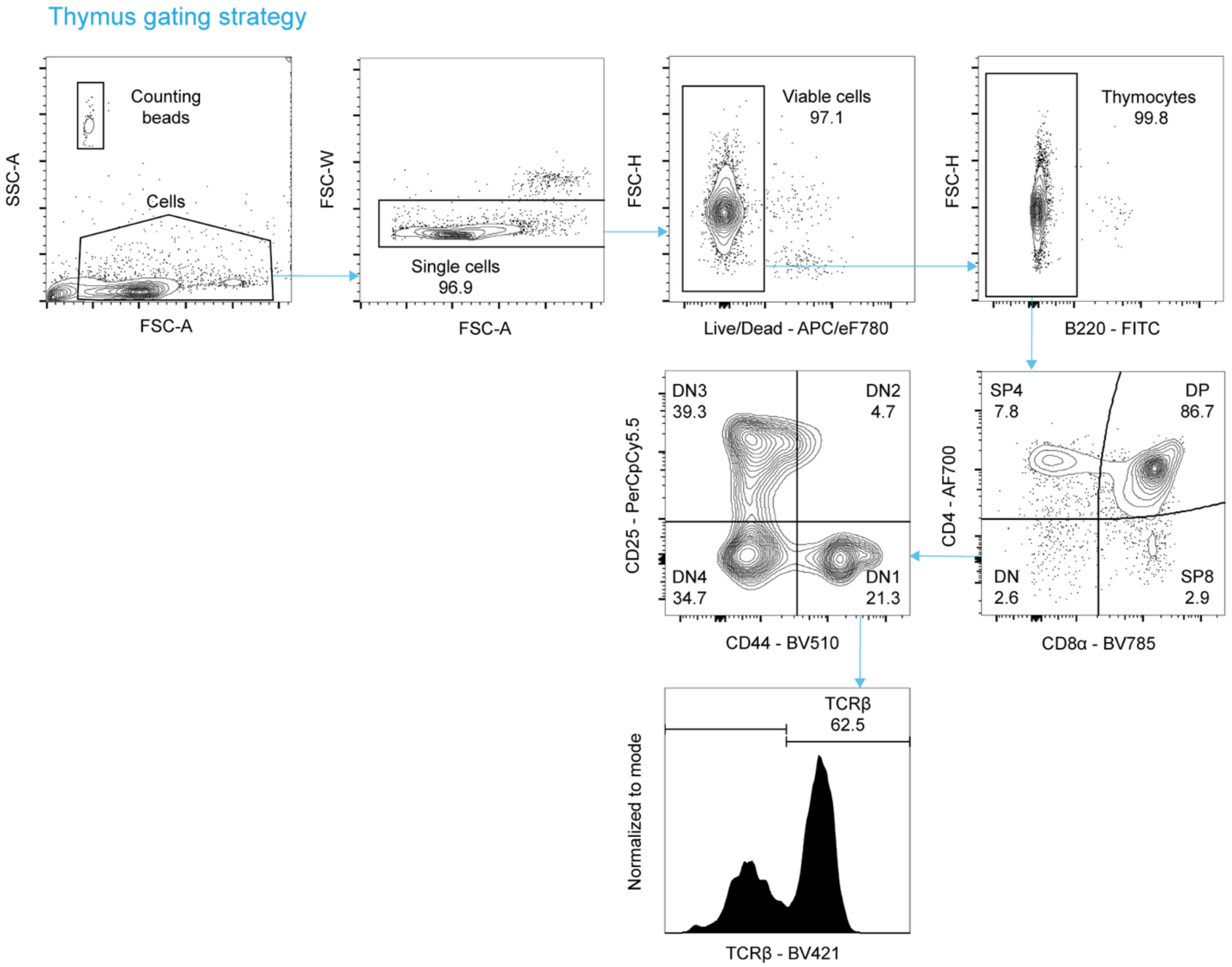

Supplemental Figure 9. Representative gating strategy for thymocytes. Representative gating strategy for thymocyte characterization.

Supplemental Tables.

Supplemental Table 1. Key resource table.

REAGENT or RESOURCE	SOURCE	IDENTIFIER
**Antibodies**		
BV785 anti-mouse CD8α (53-6.7)	BioLegend	Cat #100750; RRID: AB_2562610
AlexaFluor700 anti-mouse CD90.1 (OX-7)	BioLegend	Cat #202528; RRID: AB_1626241
PerCPCy5.5 anti-mouse CD90.1 (OX-7)	BioLegend	Cat #202516; RRID: AB_961437
FITC anti-mouse CD90.1 (OX-7)	BioLegend	Cat #202504; RRID: AB_1595653
FITC anti-mouse CD11a (2D7)	BioLegend	Cat # 101106; RRID: AB_312779
BV510 anti-mouse CD11a (2D7)	BD Biosciences	Cat #740110; RRID: AB_2739868
PE anti-mouse Vα2 (B20.1)	BioLegend	Cat #127808; RRID: AB_1134183
APC anti-mouse CD45 (30-F11)	BioLegend	Cat #103112; RRID: AB_312977
Pacific Blue anti-mouse CD45 (30-F11)	BioLegend	Cat #103126; RRID: AB_493535
Pacific Blue anti-mouse CD45.2 (104)	BioLegend	Cat #109820; RRID: AB_492872
BV421 anti-mouse CD45.2 (104)	BioLegend	Cat #109832; RRID: AB_2565511
PE/CF594 anti-mouse CD69 (H1.2F3)	BD Biosciences	Cat #562455; RRID: AB_11154217
BB700 anti-mouse CD49a (Ha31/8)	BD Biosciences	Cat #742164; RRID: AB_2861198
PE anti-mouse CD103 (2E 7)	BioLegend	Cat #121406; RRID: AB_1133989
PECy7 anti-mouse CXCR6 (SA051D1)	BioLegend	Cat #151119; RRID: AB_2721670
BV421 anti-mouse PD-1 (29F.1A12)	BioLegend	Cat #135221; RRID: AB_2562568
PE anti-mouse PD-1 (29F.1A12)	BioLegend	Cat #135206; RRID: AB_1877231
BV510 anti-mouse CD62L (MEL-14)	BioLegend	Cat #104441; RRID: AB_2561537
PE anti-mouse CX3CR1 (SA011F11)	BioLegend	Cat #149006; RRID: AB_2564315
BV421 anti-mouse IFN-γ (XMG1.2)	BioLegend	Cat #505830; RRID: AB_2563105
PECy7 anti-mouse TNF (MP6-XT22)	BioLegend	Cat #506324; RRID: AB_2256076
PE anti-mouse CD107a (1D4B)	BioLegend	Cat #121612; RRID: AB_2134487
AlexaFluor700 anti-mouse CD4 (GK1.5)	eBioscience	Cat #56-0041-82; RRID: AB_493999
FITC anti-mouse CD4 (H129.19)	BioLegend	Cat #130308; RRID: AB_1279237

BV510 anti-mouse B220 (RA3-6B2)	BioLegend	Cat #103248; RRID: AB_2650679
FITC anti-mouse B220 (RA3-6B2)	eBioscience	Cat #11-0452-82; RRID: AB_465054
AlexaFluor700 anti-mouse Ly6G (1A8)	BioLegend	Cat #127622; RRID AB_10643269
PE anti-mouse Ly6C (HK1.4)	BioLegend	Cat #128008; RRID AB_1186132
PerCpCy5.5 anti-mouse Ly6C (HK1.4)	BioLegend	Cat #128012; RRID AB_1659241
PEDazzle594 anti-mouse CD11c (N418)	BioLegend	Cat #117347; RRID: AB_2563654
PECy7 anti-mouse CD11b (M1/70)	BioLegend	Cat #101216; RRID: AB_312799
PE anti-mouse NK1.1 (PK136)	BioLegend	Cat #108708; RRID: 108708
PE anti-mouse NKp46 (29A1.4)	BioLegend	Cat #137604; RRID: AB_2235755
BV510 anti-mouse CD44 (IM7)	BioLegend	Cat #103043; RRID: AB_2561391
PerCpCy5.5 anti-mouse CD25 (PC61)	BioLegend	Cat #102030; RRID: AB_893291
BV421 anti-mouse TCRβ (H57-597)	BioLegend	Cat #109230; RRID: AB_10933263
PE anti-mouse CD5 (53-7.3)	BioLegend	Cat #100608; RRID: AB_312737
PECy7 anti-mouse CD69 (H1.2F3)	BioLegend	Cat #104512; RRID: AB_493564
AF647 anti-mouse CD31 (MEC13.3)	BioLegend	Cat #102516; RRID: AB_2161029
APC anti-mouse Ki-67 (SolA15)	Thermo Fisher	Cat #17-5698-82; RRID: AB_2688057
FITC anti-mouse Granzyme B (GB11)	BioLegend	Cat #515403; RRID: AB_2114575
BV510 anti-mouse TIM-3 (5D12)	BD Biosciences	Cat #747625; RRID: AB_2744191
APC/eF780 fixable viability stain	BD Biosciences	Cat #565388; RRID: AB_2869673
H2-D^b $TRP1_{455-463}$	Harty lab	NA
H2-K^b $TRP2_{180-188}$	Harty lab	NA
H2-D^b $GP100_{25-33}$	Harty lab	NA
H2-D^b $NP_{366-374}$	Harty lab	NA
H2-D^b $PA_{224-233}$	Harty lab	NA
H2-K^b $OVA_{257-264}$	Harty lab	NA
a-Thy1.1 (19E12)	BioXCell	Cat #BE0214; RRID: AB_2687700
a-GR-1 (NIMP-R14)	BioXCell	Cat #BE0320; RRID: AB_2819047
a-PD-L1 (10F.9G2)	BioXCell	Cat #BE0101; RRID: AB_10949073
**Bacterial and virus strains**		
Listeria monocytogenes expressing -$OVA_{257-264}$, attenuated (ΔactA, ΔinlB-deficient)	Harty lab	NA

Listeria monocytogenes expressing -GP_{33-41}, attenuated (ΔactA, ΔinlB-deficient)	Harty lab	NA
Listeria monocytogenes expressing -$TRP2_{180-188}$, attenuated (ΔactA, ΔinlB-deficient)	Harty lab	NA
Listeria monocytogenes expressing -$PA_{224-233}$, attenuated (ΔactA, ΔinlB-deficient)	Harty lab	NA
Lymphocytic choriomeningitis virus (LCMV) strain Armstrong	Harty lab	NA
**Chemicals, peptides, and recombinant proteins**		
ACK lysis buffer	Harty lab	NA
Vitalyse	CMDG	Cat #WBL0100
Collagenase D	Sigma Aldrich	Cat #11088866001
Collagenase II	Sigma Aldrich	Cat #17101015
Liver digestion buffer	Gibco	Cat #17703034
DNase I	Sigma Aldrich	Cat #D4513-1VL
Percoll	GE Healthcare	Cat #17-0891-01
Hepes	Gibco	Cat #15630080
DPBS	Gibco	Cat #14190144
RPMI	Gibco	Cat #11875093
DMEM	Gibco	Cat #11965092
HBSS	Gibco	Cat #14025092
FACS Buffer	Harty lab	NA
0.25% Trypsin-EDTA	Gibco	Cat #25200056
TrypLE™ Express Enzyme	Gibco	Cat #12604013
Nuclease-free water	Thermo Fisher	Cat #AM9937
Brefeldin A	BioLegend	Cat #420601
24.2G Fc Block	Harty lab	NA
FACS Buffer	Harty lab	NA
Cytofix Fixation Buffer	BD Bioscience	Cat #554655
Igepal	Sigma Aldrich	Cat #56741
Tryptic Soy Broth	BD Bioscience	Cat #BA-257107.06
Methylcellulose	Sigma Aldrich	Cat #M7027
Low melting point agarose	Promega	Cat #V2111
ProLong™ Gold Antifade Mountant with DAPI	Thermo Fisher	Cat #P36935
Lipopolysaccharide	Sigma Aldrich	Cat # L8274
Triton™ X-100	Sigma Aldrich	Cat #X100
VivoGlo™ D-luciferin	Promega	Cat #P1043
Ψ UTP	Thermo Fisher	Cat #N-1019-1
5-M CTP	Thermo Fisher	Cat #N-1014-1
LipidFlex lipid formulation	PreciGenome	Cat #PG-SYN-LF1ML
SM-102 cationic lipid	MedChemExpress	Cat #HY-134541
$TRP1_{455-463}$	Global Peptide	TAPDNLGYM
$TRP2_{180-188}$	Global Peptide	SVYDFFVWL
$GP100_{25-33}$	Global Peptide	KVPRNQDWL
$OVA_{257-264}$	Global Peptide	SIINFEKL
$NP_{366-374}$	Global Peptide	ASNENMETM
$PA_{224-233}$	Global Peptide	SSLENFRAYV
GP_{33-41}	Global Peptide	KAVYNFATC
Streptavidin-APC	Thermo Fisher	Cat #S868

Streptavidin-PE	Thermo Fisher	Cat #S866
Streptavidin-FITC	Thermo Fisher	Cat #S869
Streptavidin-BV421	BioLegend	Cat #405225
**Critical commercial assays**		
CountBright™ Absolute Counting Beads, for flow cytometry	Thermo Fisher	Cat #C36950
FoxP3 / Transcription Factor Staining Kit	Tonbo / Cytek	Cat #SKU TNB-0607-KIT
CD11c Microbeads UltraPure	Miltenyi Biotec	Cat #130-125-835
ARCA T7 mRNA Synthesis Kit	NEB	Cat #E2065
RNeasy Mini Kit	Qiagen	Cat #74104
Quant-iT™ Ribogreen® Assay Kit	Thermo Fisher	Cat #R11490
**Experimental models: Cell lines**		
B16 F10 melanoma cell line	Harty lab	NA
B16-OVA F10 melanoma cell line	Lyse Norian lab (University of Alabama)	NA
B16-GP33 F10 melanoma cell line	Ryan Zander lab (University of Iowa)	NA
GL261-quad cassette-luciferase glioblastoma cell	Aaron Johnson lab (Mayo Clinic)	NA
**Experimental models: Organisms/strains**		
C57BL/6N	Charles River	000027
OT-I	Jackson Laboratories	003831
P14	Jackson Laboratories	037394
Thy1.1	Jackson Laboratories	000406
OT-I Thy1.1	Harty Lab	NA
P14 Thy1.1	Harty Lab	NA
TCRβKO	Jackson Laboratories / Butler Lab	002118
**Recombinant DNA**		
pMRNAxp mRNAExpress vector for in vitro transcription	System Bio	Cat #MR000PA-1
**Software and algorithms**		
FlowJo v10.10.1 (with Downsample and UMAP plug-ins)	FlowJo, LLC	https://www.flowjo.com/solutions/flowjo/
Xenogen Living Image software	Caliper Life Sciences	https://www.revvity.com/product/spectrum-200-living-image-v4series-1-128113
Adobe Illustrator v24.0.1	Adobe	https://www.adobe.com/products/illustrator.html
Prism 10.1.1	GraphPad Software	https://www.graphpad.com/features
**Other**		
70 μm Filters	Thermo Fisher	Cat #22-363-548
Ethicon Perma-Hand Silk Suture, Size 4-0, 18”	Thermo Fisher	Cat #683G
Ethicon Coated Vicryl Suture, Size 5-0, 18”	Thermo Fisher	Cat #J493G
Vetbond	3M	Cat #B00016067
Hamilton 25 μL Microliter Syringe Model 702 N, Cemented Needle, 22s gauge, 2 in, point style 2	Hamilton	Cat #80400

BD 1 mL Micro-Fine™ IV Insulin Syringe	Thermo Fisher	Cat #14-829-1A
BD 1 mL TB Syringe	Thermo Fisher	Cat #309623
Vernier calipers	Thermo Fisher	Cat #S90343

Supplemental Table 2. mRNA vaccine amino acid and coding sequences.

**Vaccine components**	
Coding sequences	Kozak sequence: GCCACC<br>Ub-A76:<br>ATGCAGATCTTCGTGAAGACCCTGACCGGCAAGACCATCACCCTGGAG<br>GTGGAGCCCAGTGACACCATCGAGAACGTGAAGGCCAAGATCCAGGA<br>TAAAGAGGGCATCCCCCCTGACCAGCAGAGGCTGATCTTTGCCGGCA<br>AGCAGCTGGAAGATGGCCGCACCCTCTCTGATTACAACATCCAGAAAG<br>AGTCAACCCTGCACCTGGTCCTCCGTCTGAGGGGTGCC<br>Flexible linker: GTAGGAAAAGGTGGTTCAGGAGGC<br>Proteasome recognition spacers: GCTGCTTAT<br>TRP1$_{455-463}$: ACTGCTCCAGACAACCTGGGATACATG<br>TRP2$_{180-188}$: AGTGTTTATGATTTTTTTGTGTGGCTC<br>GP100$_{25-33}$: AAAGTACCCAGAAACCAGGACTGGCTT<br>OVA$_{257-264}$: AGTATAATCAACTTTGAAAAACTG<br>NP$_{366-374}$: GCTTCCAATGAAAATATGGAGACTATG<br>PA$_{224-233}$: TCCAGCCTTGAAAATTTTAGAGCCTATGTG<br>GP$_{33-41}$: AAAGCTGTGTACAATTTCGCCACCATG
Amino acid sequence	Ub-A76:<br>MQIFVKTLTGKTITLEVEPSDTIENVKAKIQDKEGIPPDQQRLIFAGKQLEDG<br>RTLSDYNIQKESTLHLVLRLRGA<br>Flexible linker: VGKGGSGG<br>Proteasome recognition spacers: AAY<br>TRP1$_{455-463}$: TAPDNLGYM<br>TRP2$_{180-188}$: SVYDFFVWL<br>GP100$_{25-33}$: KVPRNQDWL<br>OVA$_{257-264}$: SIINFEKL<br>NP$_{366-374}$: ASNENMETM<br>PA$_{224-233}$: SSLENFRAYV<br>GP$_{33-41}$: KAVYNFATM
**UbMel-OVA vaccine**	
Coding sequence	GCCACCATGCAGATCTTCGTGAAGACCCTGACCGGCAAGACCATCACC<br>CTGGAGGTGGAGCCCAGTGACACCATCGAGAACGTGAAGGCCAAGAT<br>CCAGGATAAAGAGGGCATCCCCCCTGACCAGCAGAGGCTGATCTTTG<br>CCGGCAAGCAGCTGGAAGATGGCCGCACCCTCTCTGATTACAACATCC<br>AGAAAGAGTCAACCCTGCACCTGGTCCTCCGTCTGAGGGGTGCCGTA<br>GGAAAAGGTGGTTCAGGAGGCGCTGCTTATACTGCTCCAGACAACCTG<br>GGATACATGGCTGCTTATAGTGTTTATGATTTTTTTGTGTGGCTCGCTG<br>CTTATAAAGTACCCAGAAACCAGGACTGGCTTGCTGCTTATAGTATAAT<br>CAACTTTGAAAAACTGGCTGCTTATTAG

Amino acid sequence	MQIFVKTLTGKTITLEVEPSDTIENVKAKIQDKEGIPPDQQRLIFAGKQLEDG<br>RTLSDYNIQKESTLHLVLRLRGAVGKGGSGGAAYTAPDNLGYMAAYSVYD<br>FFVWLAAYKVPRNQDWLAAYSIINFEKLAAY
**UbFlu-GP33 vaccine**	
Coding sequence	GCCACCATGCAGATCTTCGTGAAGACCCTGACCGGCAAGACCATCACC<br>CTGGAGGTGGAGCCCAGTGACACCATCGAGAACGTGAAGGCCAAGAT<br>CCAGGATAAAGAGGGCATCCCCCCTGACCAGCAGAGGCTGATCTTTG<br>CCGGCAAGCAGCTGGAAGATGGCCGCACCCTCTCTGATTACAACATCC<br>AGAAAGAGTCAACCCTGCACCTGGTCCTCCGTCTGAGGGGTGCCGTA<br>GGAAAAGGTGGTTCAGGAGGCGCTGCTTATGCTTCCAATGAAAATATG<br>GAGACTATGGCTGCTTATTCCAGCCTTGAAAATTTTAGAGCCTATGTG<br>GCTGCTTATAAAGCTGTGTACAATTTCGCCACCATGGCTGCTTATTAG
Amino acid sequence	MQIFVKTLTGKTITLEVEPSDTIENVKAKIQDKEGIPPDQQRLIFAGKQLEDG<br>RTLSDYNIQKESTLHLVLRLRGAVGKGGSGGAAYASNENMETMAAYSSLE<br>NFRAYVAAYKAVYNFATMAAY
